# A new microfluidic model to study dendritic remodeling and mitochondrial dynamics during axonal regeneration of adult zebrafish retinal neurons

**DOI:** 10.3389/fnmol.2023.1196504

**Published:** 2023-06-15

**Authors:** Annelies Van Dyck, Luca Masin, Steven Bergmans, Giel Schevenels, An Beckers, Benoit Vanhollebeke, Lieve Moons

**Affiliations:** ^1^Neural Circuit Development and Regeneration Research Group, Animal Physiology and Neurobiology Division, Department of Biology, Leuven Brain Institute, KU Leuven, Leuven, Belgium; ^2^Laboratory of Neurovascular Signaling, Department of Molecular Biology, ULB Neuroscience Institute, Université libre de Bruxelles, Gosselies, Belgium

**Keywords:** axonal regeneration, zebrafish, *in vitro*, microfluidics, mitochondria, dendrites

## Abstract

Unlike mammals, adult zebrafish are able to fully regenerate axons and functionally recover from neuronal damage in the mature central nervous system (CNS). Decades of research have tried to identify the mechanisms behind their spontaneous regenerative capacity, but the exact underlying pathways and molecular drivers remain to be fully elucidated. By studying optic nerve injury-induced axonal regrowth of adult zebrafish retinal ganglion cells (RGCs), we previously reported transient dendritic shrinkage and changes in the distribution and morphology of mitochondria in the different neuronal compartments throughout the regenerative process. These data suggest that dendrite remodeling and temporary changes in mitochondrial dynamics contribute to effective axonal and dendritic repair upon optic nerve injury. To further elucidate these interactions, we here present a novel adult zebrafish microfluidic model in which we can demonstrate compartment-specific alterations in resource allocation in real-time at single neuron level. First, we developed a pioneering method that enables to isolate and culture adult zebrafish retinal neurons in a microfluidic setup. Notably, with this protocol, we report on a long-term adult primary neuronal culture with a high number of surviving and spontaneously outgrowing mature neurons, which was thus far only very limitedly described in literature. By performing time-lapse live cell imaging and kymographic analyses in this setup, we can explore changes in dendritic remodeling and mitochondrial motility during spontaneous axonal regeneration. This innovative model system will enable to discover how redirecting intraneuronal energy resources supports successful regeneration in the adult zebrafish CNS, and might facilitate the discovery of new therapeutic targets to promote neuronal repair in humans.

## 1. Introduction

Neurodegenerative diseases or brain trauma often result in irreversible impairments in the adult mammalian central nervous system (CNS), as damaged neurons cannot be repaired or replaced, and the few surviving neurons fail to regrow their axons beyond the injury site (Fawcett, 2020; Otsuki and Brand, 2020; Varadarajan et al., 2022). Despite many years of research, functional recovery is still not possible in the damaged or diseased mammalian CNS, and discovery of new therapeutic targets thus remains crucial (Williams et al., 2020; Varadarajan et al., 2022). One striking gap in axonal regeneration research is that the contribution of cellular and molecular processes in the dendrites has been consistently overlooked, even though dendrites form an essential component of the neuronal circuitry, and defects in the dendritic arbors often precede axonal degeneration in neurodegenerative diseases (Kulkarni and Firestein, 2012; Della Santina et al., 2013; Agostinone et al., 2018; Peterson and Benowitz, 2018; Claes et al., 2021). Over the years, several pro-regenerative mechanisms have been identified by studying retinal ganglion cells (RGCs) and optic nerve regeneration (Berry et al., 2019; Williams et al., 2020). One key advantage of the eye-to-brain pathway is that axons and dendrites of RGCs are spatially separated. This segregated anatomy enables to independently investigate the responses to injury that occur in each neuronal compartment and to determine the intraneuronal processes that are contributing to potential circuit repair.

While sharing many regeneration-associated genes and pathways with mammals, zebrafish (*Danio rerio*) are capable of spontaneously repairing damaged neurons in their adult CNS (Fleisch et al., 2011; Becker and Becker, 2014; Rasmussen and Sagasti, 2016). Because of this robust regenerative capacity, they have become a well-established and powerful model in the regenerative field. Upon optic nerve crush (ONC) injury, adult zebrafish RGCs survive and successfully reconnect with their target neurons in the optic tectum, resulting in full visual recovery (Becker and Becker, 2014; Dhara et al., 2019). Using this model, we previously uncovered an antagonistic axon-dendrite interplay, wherein RGC dendritic shrinkage is evoked immediately after ONC injury, prior to axonal regrowth, and dendrites regenerate only after axonal repair is completed. Moreover, inhibiting this retraction resulted in disturbed axonal regeneration (Beckers et al., 2019). These findings suggest that dendritic shrinkage may be an important stimulant for effective neuronal circuit restoration.

This observed interaction may result from an energy restriction that prevents simultaneous regrowth of axons and maintenance of dendrites. Indeed, axonal regeneration is a highly energy-demanding process, and the diffusion of adenosine triphosphate (ATP) is limited within the long, branched architecture of mature neurons (Patrón and Zinsmaier, 2016). Moreover, mitochondria, the main energy suppliers of neurons, become depolarized after axonal injury, further limiting the availably of energy to sustain regrowth (Cavallucci et al., 2014; Zhou et al., 2016). Emerging evidence from *in vitro* and *in vivo* studies in spontaneously as well as experimentally induced regenerating models suggests that increased mitochondrial mobility is essential to enable successful axonal regrowth (Mar et al., 2014; Han et al., 2016; Zhou et al., 2016; Cartoni et al., 2017; Xu et al., 2017; Cheng et al., 2022). In line with this, we recently discovered that both the distribution and morphology of mitochondria change within RGCs during the different phases of axonal and dendritic regrowth induced by ONC injury in adult zebrafish (Beckers et al., 2023c).

However, as numerous RGC axons are tightly packed together in the optic nerve and not all RGCs respond in the same time frame to optic nerve injury, bulk analyses of mitochondrial densities may mask true bioenergetic changes of individual cells. Also, *postmortem* samples merely show a one-time snapshot, and thus do not provide any information on the true dynamics of mitochondria. A real-time, compartment-specific study at single-cell level is therefore key to truly uncover how energy allocation shifts among distinct compartments of individual neurons. Even though the segregated anatomy of the retinotectal system enables to study injury responses in adult RGC somata, dendrites and axons separately, experimental designs that enable compartment-specific gene/protein manipulation in adult neurons remain undocumented *in vivo.* Moreover, in contrast to larvae, adult zebrafish are not transparent, thereby hampering the visualization of the entire RGC architecture *in vivo*.

The use of primary neuronal cultures provides an opportunity to study intraneuronal mechanisms at higher spatiotemporal resolution. Despite this, treatment of individual neuronal compartments remains challenging in most conventional *in vitro* setups. In this view, compartmentalized microfluidic devices (MFDs) have become a powerful research tool to overcome these hurdles. Their unique design, with two fluidically-isolated compartments that physically separate axons from cell bodies and dendrites, (1) mimics *in vivo* microenvironments in a simplified manner, (2) enables axotomy and (3) allows spatially and temporally controlled treatment of different neuronal compartments (Taylor et al., 2005, 2010; Zhou et al., 2016). More recently, they have been used to study axonal transport, mitochondrial trafficking and bioenergetics in regeneration-induced primary neurons (Zhou et al., 2016; Han et al., 2020; Cheng et al., 2022; Huang and Sheng, 2022).

In this study, we combine these MFDs with state-of-the-art live cell imaging technologies in spontaneously regenerating adult zebrafish RGCs to establish a novel *in vitro* platform that allows to investigate dendritic remodeling and the reallocation of mitochondria in the different neuronal compartments during spontaneous, injury-induced axonal regrowth of individual adult zebrafish neurons. This new model system can be exploited to gain a better understanding of the intraneuronal processes driving functional circuit repair and to uncover new targets for future regenerative therapies in the injured mammalian CNS.

## 2. Animals, materials and equipment

### 2.1. Animals

5-to 9-month-old adult zebrafish (*D. rerio*) of both sexes from the following lines are used: wild type AB (WT) fish, *Tg(Tru.gap43:GFP)^mil1^* (gap43) fish, in which GFP is expressed under the control of the *gap43* promotor, labeling outgrowing neurons (i.e., RGCs) (Udvadia, 2008; Diekmann et al., 2015), and *Tg(Tru.gap43:mitoEGFP-2A-tagRFP-CAAX)^ulb17^* (gapmito) fish, in which GFP is fused to the mitochondrial targeting sequence of the zebrafish cytochrome c oxidase subunit 8A (cox8a) and TagRFP to a membrane localization motif (CAAX), both under control of the same *gap43* promotor, thereby visualizing, respectively, mitochondria and plasma membranes of outgrowing neurons. The latter transgenic line has been generated using the Tol2 transposase system as previously described (Kwan et al., 2007). Briefly, the pT2-Tru.gap43:mls-EGFP-2A-tagRFP-CAAX plasmid was generated by cloning the 3.6 kb pufferfish *gap43* promotor element upstream of the mls-EGFP-2A-tagRFP-CAAX ORF (Udvadia, 2008; Verreet et al., 2019). All fish lines are bred and maintained under standard conditions (28°C, 14/10-h light/dark cycle) and procedures performed in accordance with the 2010/63/EU European Communities Council Directive and approved by the KU Leuven Ethical Committee for Animal Experiments.

### 2.2. List of reagents, materials and equipment

#### 2.2.1. Optic nerve crush and retinal dissection/isolation

- Tricaine stock solution: Dissolve 2,1 g tricaine powder (MS-222, Sigma Aldrich) in 685.3 mL ultrapure water with 14.7 mL (20.6 mM) Tris–HCl to a final concentration of 0.3% (w/v) and adjust to pH 7.0. Store in the dark at 4°C for up to 3 months.
- Anesthetic solution: Dilute 6.7 mL of tricaine stock solution in 93.3 mL system water to a final concentration of 0.02% tricaine (w/v). Store at 4°C for up to 1 week.
- Euthanasia solution: Dilute 33.3 mL tricaine stock solution in 66.7 mL system water to a final concentration of 0.1% tricaine (w/v). Sterilize by passing through a 0.22 μm filter and store at 4°C for up to 1 week.
- Fish culture medium: prepare a supplement solution by adding to 1 L of MilliQ water, 0.18 g CaCl2 (1.26 mM), 7.6 g HEPES (32 mM) and 1.98 g D-glucose (10 mM). Adjust to pH 7.4.
  For 500 mL of fish medium, add 62.5 mL of the supplement solution, 10 mL fetal bovine serum (10,270,106, Gibco), 10 mL Penicillin/Streptomycin (P/S; 15,140,122, Gibco) and 250 μL Amphotericin B (15,290,018, Gibco) to 437.5 mL Leibovitz’s L-15 medium (11,415,064, Gibco), to obtain a final concentration of 12,5% (v/v) supplements, 2% (v/v) P/S, and 0.05% (v/v) Amphotericin B. Filter-sterilize (0.22 μm) and store at 4°C for up to 1 month (Diekmann et al., 2015).
- 70% (v/v) ethanol in ultrapure water
- Forceps (Dumont #5, 11,252–20, Fine Science Tools)
- Vannas Spring Scissors (15000–08, Fine Science Tools)
- Disposable injection needle (30G 0.30 × 12 mm)
- Disposable winged needle (21G 0.8 × 19 mm)
- Sterile 10 cm ⌀ polystyrene petri dishes
- Injection syringe (25 mL)
- Eppendorf tubes (2 mL)
- Sterile gauze
- Hot bead sterilizer (240°C; 1,800,045, Fine Science Tools)
- Stereo microscope with an upper light source (e.g., S6E Stereo Microscope, Leica)
- Horizontal flow cabinet (e.g., HELIOS, Angelantoni Life Science)

#### 2.2.2. Assembly of microfluidic devices

- Ultrapure distilled water (10,977,035, Gibco)
- Sterile Dulbecco’s Phosphate Buffered Saline (DPBS) (14,190,144, Gibco)
- XC Pre-Coat solution (Xona Microfluidics)
- 68% (v/v) nitric acid (20406.292, VWR Chemicals)
- 70% Ethanol
- #1.5 thickness glass coverslips (25 × 75 mm; 10,812, IBIDI)
- Polydimethylsiloxane (PDMS) microfluidic chambers with 450 μm microgroove length: single open compartment (SOC450) and Standard Neuron Device (SND450) (Xona Microfluidics)
- Sterile 10 cm ⌀ polystyrene petri dishes
- Sterile 10 cm ⌀ glass petri dishes
- Scotch tape
- Sterile forceps
- Laboratory beaker (500 mL)
- Whatman paper (2105841, Sigma-Aldrich)
- Plasma cleaner setup: Expanded Plasma Cleaner, Vacuum Gauge, Economy Dry Oxygen Pump (PDC002CE, Harrick Plasma Inc.).
- Biosafety cabinet (Labculture class II A2 BSC, ESCO)
- Drying oven (50°C/180°C; Forced Air Safety Oven, 52201–214, VWR)
- Fume hood
- Magnetic hotplate stirrer (100°C; 442–0185; VWR)

#### 2.2.3. Adult zebrafish microfluidic cultures

- Fish culture medium (Section 2.2.1)
- Complete Neurobasal Medium (CNB): For 50 mL of CNB medium, add 1 mL B-27 supplement (17,504,044, Gibco), 500 μL L-glutamine (25,030,081, Gibco), 125 μL HEPES (15,630,056, Gibco), 1 mL P/S and 25 μL Amphotericin B to 47.5 mL Neurobasal medium (21,103,049, Gibco), to obtain a final concentration of 2% (v/v) B-27, 1% (v/v) L-glutamine, 2% (v/v) P/S and 0.25% (v/v) Amphotericin B. Filter-sterilize (0.22 μm) and store at 4°C for up to 2 weeks.
- Poly-L-Lysine solution (PLL): Dissolve 5 mg of PLL powder (P6282, Sigma-Aldrich) in 15 mL MilliQ to a final working concentration of 333 μg/mL and store in 1 mL aliquots at 4°C.
- Laminin stock solution: Dilute Laminin (23,017,015, Gibco) in sterile DPBS to a final concentration of 1 mg/mL and store in 1 mL aliquots at −20°C.
- Papain stock solution: To 1 L of MilliQ water, add 0.409 g (1.1 mM) EDTA, 0.966 g (5.5 mM) Cysteine-HCL and 4.67 μL (0.067 mM) B-Mercaptoethanol. Dissolve lyophilized papain powder (LS003118, Worthington) to a final concentration of 800 U/mL. Store in 20 μL aliquots at −20°C for up to 3 months.
- DNase stock solution: Dissolve lyophilized powder (LK003170, Worthington) in sterile DPBS to a final concentration of 2000 U/mL. Store in 10 μL aliquots at −20°C for up to 3 months.
- Trypan blue solution (T8154, Sigma-Aldrich)
- 70% ethanol
- Sterile pipettes and tips (P10-P1000)
- Eppendorf tubes (1.5, 2 mL)
- Low Adhesion microcentrifuge tubes (1.5 mL)
- Conical tubes (5, 50 mL)
- PVDF syringe filter unit (0.22 μm pore size)
- Sterile Flat-Bottom polystyrene 96-well plate (267,544, Thermo Fisher)
- Water bath (30°C, 37°C)
- Automated cell counter (TC20, Biorad)
- Inverted fluorescence microscope (e.g., DMIL Led microscope with LAS v4.6 software, Leica)
- Vacuum aspirator pump (VP 86, VWR)
- Swinging bucket centrifuge (200 rcf, 30°C; e.g., 5,702 R, Eppendorf)
- Tissue culture incubator (30°C, 5% CO_2_; e.g., MCO-5 AC, Sanyo)
- Biosafety cabinet (Labculture class II A2 BSC, ESCO)

#### 2.2.4. (Time-lapse live cell) microscopy

- MitoTracker™ Red FM stock solution: Dissolve Mitotracker Red powder (M22425, Invitrogen) in Dimethylsulfoxide (DMSO) to a final concentration of 1 mM. Store aliquots of 10 μL at −20°C for up to 1 year.
- Calcein blue, AM stock solution: Dissolve the Calcein (C1429, Invitrogen) in DMSO to a final stock concentration of 2 mM. Store in aliquots of 10 μL at −20°C for up to 3 months.
- Vybrant DiI (V22885, Invitrogen): Store at −20°C.
- Inverted confocal microscope equipped with 488 and 561 nm lasers and corresponding excitation and emission filters (LSM900 with Airyscan2 and Zen Pro Software, Zeiss).
- Heat and gas adjustable microscopic incubation chamber (equilibrated at 30°C and 5% CO_2_; STXF stage top incubator, Tokai Hit)
- ImageJ software with KymographBuilder plugin (Fiji)

#### 2.2.5. Fixation and immunostaining

- Phosphate buffered saline (PBS): 137 mM NaCl, 2.7 mM KCl, 10 mM Na_2_HPO_4_, 1.8 mM KH_2_PO_4_ in MilliQ. Adjust to pH 7.4.
- 4% (v/v) Paraformaldehyde (PFA): Dissolve 4 g PFA in 100 mL PBS, filter (0.22 μm) and store at 4°C for up to 1 week.
- PBS-Triton X-100 (PBST): Add 100 μL Triton X-100 to 50 mL PBS to a final working concentration of 0.2% (v/v). Store at 4°C.
- 4′,6-diamidino-2-phenylindole (DAPI) (32,670, Sigma Aldrich): Dilute in PBS to a final stock concentration of 0.1% and store in the dark at −20°C.
- Primary antibodies: mouse anti-Acetylated tubulin (T6793, Sigma Aldrich), mouse anti-Map2ab (M1406, Sigma Aldrich), mouse anti-Tau (MAB3420, Sigma Aldrich): Store in the dark at −20°C.
- Donkey-anti-mouse Alexa Fluor-conjugated secondary antibody (DAM-Alexa 647; A-31571, Thermo Fisher): Store in the dark at 4°C.
- Bovine Serum Albumin (BSA) Fraction V (A2244, AppliChem).

## 3. Methods

**Figure 1:**
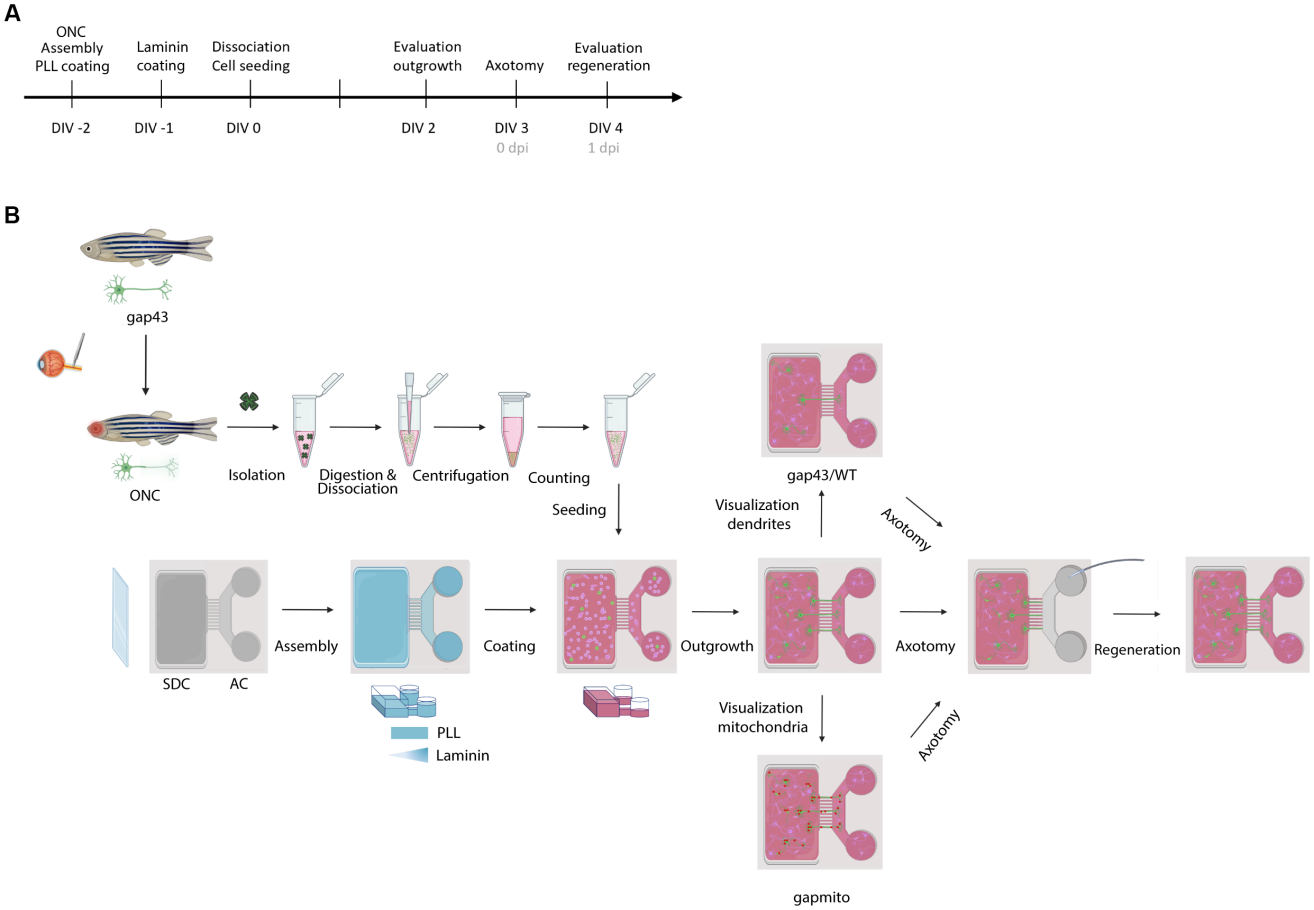
Schematic representation of the experimental setup. **(A)** Timeline of the performed procedures. **(B)** Two days before culturing (DIV-2), fish are subjected to ONC to prime RGCs and improve their outgrowth in culture. In addition, MFDs are assembled by adhering the PDMS top part on a cleaned (acid treated) glass cover slip using plasma treatment. Next, MFDs are coated overnight with consecutively PLL (DIV-2) and Laminin (DIV-1). For both coatings, a volumetric gradient is used to ensure coating of the grooves. Additionally, a Laminin concentration gradient (from the AC to the SDC) is used to promote axonal outgrowth toward the AC. The next morning, adult zebrafish retinas are isolated, followed by an enzymatic and mechanical dissociation to obtain a single cell suspension. To remove dead cells and debris, the suspension is centrifuged and resuspended in fresh fish medium, and retinal cells are seeded in the SDC of an open compartment MFD (SOC450). To stimulate outgrowth of RGC axons toward the AC, a volumetric gradient from the SDC to the AC is created and conditioned retinal cells are also seeded in the AC. Cells are cultured at 30°C and 5% CO_2_ and monitored daily. At DIV 2, RGC axonal outgrowth can be examined. At DIV 3, a vacuum-assisted axotomy is performed, whereafter axonal regeneration can be evaluated. To visualize the regenerative process and characterize mitochondrial dynamics or dendritic remodeling, respectively, *Tg(Tru.gap43:GFP)^mil^* (gap43), *Tg(Tru.gap43:mitoEGFP-2A-TagRFP-CAAX)*^ulb17^ (gapmito) or a sparsely-labeled, mixed culture of wild type (WT) and (gap43) retinal neurons are seeded in the SDC. AC, axonal compartment; DIV, *days in vitro*; MFD, microfluidic device; ONC, optic nerve crush; PLL, Poly-L-lysine; RGC, retinal ganglion cell; SDC, somatodendritic compartment.

A schematic overview of all experimental procedures and the corresponding timeline are depicted in Figure 1.

Prepare and filter all stock solutions and culture media on beforehand in a sterile environment, unless indicated otherwise. Recipes and storage conditions are described in Section 2.

### 3.1. Optic nerve crush – 5 min (DIV -2)

ONC is a frequently used injury model to induce axonal damage to RGC axons. Here, an ONC injury is performed 2 days before isolation and seeding of retinal neurons (*day in vitro-2*, DIV-2), as a conditioning lesioning (CL) to prime the regenerative capacities of the RGCs and increase the number of outgrowing axons in culture. The ONC procedure is performed as previously described (Van Dyck et al., 2021; Dhara and Udvadia, 2023; Beckers et al., 2023a,b,c).

1. Use a fishnet to transfer the fish into the anesthetic solution. Wait until balance is lost and gill movements have stopped completely.
2. Position the fish on a moist tissue paper soaked in anesthetic solution under a stereo microscope.
3. To crush the left eye, position the fish on its right lateral side (left side facing up) and cover the gills with another tissue paper soaked in anesthetic solution.
4. Remove the dermal layer of the cornea using sterile forceps and gently lift the eye out of its socket to expose the optic nerve.
5. Crush the optic nerve at a distance of 500 μm from the optic nerve head by applying a constant pressure for 10 s using another pair of sterile forceps.
6. Gently push the eye back in the socket with the flat edge of the forceps.
7. Awake the fish by moving it lightly in the water of a new tank to provide a stream of fresh water over its gills.
8. House the injured fish in a separate recovery tank and monitor it daily.

#### Notes:

- Ensure that the ophthalmic artery remains unharmed as bleeding might affect the regeneration process. Fish that show signs of bleeding should be removed from the experiment.

### 3.2. Preparation of MFDs – 2 days

A fully assembled MFD consists of a PDMS top part tightly sealed together with a glass coverslip to create a closed environment (Taylor et al., 2005, 2010). Here, two different microfluidic designs are used, both with a somatodendritic and axonal compartment (SDC and AC) that are connected via microgrooves of 450 μm in length. In the SND450 device (Xona Microfluidics), each compartment consists of two round wells connected by a smaller channel. This design is commonly used in regenerative studies (Taylor et al., 2005, 2010; Park et al., 2006; Cartoni et al., 2017; Nagendran et al., 2018). In the SOC450 device (Xona Microfluidics), the SDC entails one big well.

#### 3.2.1. Acid treatment of glass coverslip – 24 h

To remove grease and impurities, glass coverslips are treated with nitric acid. This procedure can be performed on a large batch of cover slips, which can be stored for up to 3 months.

1. In a fume hood, pour 200 mL of nitric acid into a glass beaker (500 mL).
2. Heat up to 100°C while gently stirring on a magnetic hot plate.
3. Wait for the solution to boil before carefully adding the coverslips into the solution. Assure that all coverslips are submerged and minimize contact between separate coverslips.
4. Cover the beaker with thin foil and incubate for 1 h in a fume hood.
5. Turn the heater off and carefully discard the nitric acid.
6. Rinse the coverslips 3x thoroughly with MilliQ water and incubate overnight during the last washing step.
7. The next day, rinse 3x with MilliQ and leave to air dry on a clean Whatman paper in a fume hood. Do not overlay the coverslips.
8. Transfer the coverslips in a glass petri dish, cover with thin foil and bake for 4 h at 180°C in a drying oven.
9. Store the coverslips in a sterile environment before use.

##### Notes:

- Nitric acid is highly corrosive, always handle with caution and perform this step in a fume hood using proper protective equipment.

#### 3.2.2. Assembly of MFDs – 3 h (DIV-2)

1. Clean the PDMS top part with scotch tape to remove any dust or debris that might cause incomplete assembly and subsequent leakage.
2. Afterwards, immerse both the top part and a cleaned glass coverslip in 70% ethanol.
3. Leave to air dry for >1 h on a disinfected Whatman paper inside a biosafety cabinet with running airflow.
4. When completely dry, place the PDMS top part (patterned side facing upwards) and glass coverslip inside the plasma cleaner chamber. Keep a maximal distance between both parts.
5. Turn on the plasma cleaner and vacuum pump and wait until the pressure inside the plasma cleaner drops below 350 mTorr.
6. Switch to the highest Radio Frequency (RF) mode to initiate plasma formation. A bright violet color should be visible.
7. After 60 s, stop the process by turning off the RF switch, plasma cleaner and vacuum pump in this order, and slowly evacuate the plasma cleaner inner chamber until atmospheric pressure is reached (730 Torr) (Park et al., 2006; Lenoir et al., 2021).
8. Assemble the MFD as soon as possible after plasma treatment, as surfaces do not stay activated for a long time. Hereto, carefully place the PDMS top part (patterned side down) onto the coverslip and apply light pressure. Take note to not only seal the outer borders, but also the parts around the microgrooves. From this point on, always lift the MFD via the glass coverslip.
9. To strengthen the bond, bake the MFD in a sterile glass petri dish for 45 min at 50°C in a drying oven.
10. Expose the MFD to UV light for 30 min and transfer it to a sterile petri dish.
11. Store the assembled MFDs for up to 1 week or continue with the coating steps for immediate use (Section 3.2.2).

##### Notes:

- Use sterilized forceps to handle the glass coverslips and PDMS top part, and always keep the patterned side facing up.
- Because of the live cell imaging purposes, we here describe irreversible bonding using plasma treatment. Reversible bonding, in which the PDMS and glass slide are tightly pressed together without the use of plasma, allows for more easy staining and reuse of the top part, but comes with a higher chance of leakage and detachment (Park et al., 2006).
- As an alternative to the SND450 MFDs, a ready-to-use, pre-assembled setup exists (XC450, Xona Microfluidcs) which bypasses the need for assembly with a glass coverslip. We opted not to use them here, as open compartment MFDs are not available in this pre-assembled conformation.

#### 3.2.3. Coating of MFDs – 2 days

##### 3.2.3.1. SND450

To promote attachment and outgrowth of neurons, MFDs are coated with PLL and Laminin. To facilitate coating in the channels and microgrooves and avoid the creation of air bubbles, devices are first rinsed with XC Pre-Coat solution.

1. Add a total of 100 μL Pre-Coat into the SDC, by first administering 50 μL to the top well and allowing the fluid to flow through the channel and reach the bottom well, before adding an additional 50 μL into the bottom well. If needed, pipette a few times back and forth to push the solution through the channel. Avoid air bubbles as this will result in incomplete coating.
2. In a similar way, add 200 μL Pre-Coat to the AC (100 to the top, and 100 to the bottom well). This volume difference between the SDC and AC will create a hydrostatic pressure that will assure that the Pre-Coat will reach the full length of the microgrooves (Park et al., 2006; Taylor et al., 2010).
3. Immediately remove the Pre-Coat from both the SDC and AC wells. Take care not to remove any fluid from the main channels to avoid air bubble formation.
4. Rinse 3x with DPBS.
5. Using the same practices as described for the Pre-Coat, add 100 μL PLL to the SDC, and 200 μL PLL to the AC.
6. Incubate overnight at 4°C.
7. The next day, use a microscope to confirm that there is no leakage before continuing.
8. Aspirate the PLL from the SDC and AC wells (but not channels) and incubate with DPBS for 1 h at 30°C.
9. Wash 2x with DPBS.
10. Coat the SDC with 100 μL Laminin diluted 1:80 in sterile DPBS, and the AC with 200 μL Laminin diluted 1:40 in sterile DPBS. In combination with the volumetric differences, this asymmetric coating step generates a concentration gradient of Laminin from the AC to the SDC that will stimulate axonal outgrowth toward the AC (Virlogeux et al., 2018; Lenoir et al., 2021; Shekari and Fahnestock, 2022).
11. Incubate overnight at 4°C.

##### 3.2.3.2. SOC450

Perform all steps similarly and with the same volumes as explained for the SND450 MFD (3.2.2.1). However, as the SDC consists of one big well instead of two wells and a channel, evenly distribute 100 μL (Pre-)coating solution directly over the total surface of this open compartment, before adding an additional 200 μL to the AC (100 to the top well, and 100 to the bottom well).

###### Notes:

- Never remove fluid from the channel during any of the coating, washing, seeding, or staining steps to avoid air bubble formation and subsequent obstruction of the channels.
- Thaw and keep aliquots of Laminin stock solutions at 4°C to avoid formation of a gel.
- Always use MFDs within 2 days after Laminin coating and store at 4°C before use.

### 3.3. Primary retinal culture – 2.5 h (DIV 0)

#### 3.3.1. Preparation steps – 30 min

1. On the day of seeding, carefully remove the Laminin coating from the MFD and replace with fresh fish culture medium.
2. Pre-incubate the MFD at 30°C in the tissue cell incubator until the moment of seeding.
3. Fill 1 conical vial (50 mL, washes and seeding) and 1 Eppendorf tube (2 mL, collection of retinas) with fish culture medium and preheat at 30°C in a water bath.
4. In another Eppendorf tube (2 mL), prepare the pre-digestion solution by adding 5 μL DNase (2000 U/mL) to 975 μL of fish culture medium and preheat at 30°C in a water bath.
5. Thaw 1 aliquot of 20 μL of papain stock solution and preheat at 37°C in a water bath for a maximum of 30 min before digestion.
6. Organize the horizontal flow by disinfecting the dissection bench, as well as all equipment and tools that will be transferred into the hood using 70% ethanol, and sterilize all dissection material in a hot bead sterilizer for 10 s at 240°C.

##### Notes:

- Perform all procedures in a sterile environment and with filter-sterilized solutions to minimize contamination risk.
- Warm up all media and solutions to 30°C before use, unless indicated otherwise.
- Here, all steps are described for 4 fish retinas. All volumes and procedures should be calculated for the total number of retinas used in each experiment.

#### 3.3.2. Retinal dissection and isolation – 30 min

A schematic overview of the retinal isolation procedure is depicted in Supplementary Figure S1.

1. Euthanize the fish using a filtered euthanasia solution and place it on a sterile surgical gauze under the stereo microscope, with the crushed eye facing upwards.
2. Using a sharp needle, puncture a small hole in the dorsal side of the crushed eye.
3. Cut away the sclera and cornea using small scissors and remove the lens to expose the retina.
4. Using a winged needle connected to a syringe filled with fish medium, carefully blow sterile fish medium in between the retina and the surrounding tissues to disconnect the retina and remove the pigmented epithelium as much as possible.
5. Collect the retina by carefully cutting the optic nerve just below the optic nerve head and lifting the retina with a pair of blunt forceps.
6. Transfer the retina into the 2 mL Eppendorf tube filled with preheated fish culture medium.
7. To preserve fluorescence in the transgenic tissues, keep the tube in the dark (thin foil) until the total pool of retinas is collected.

##### Notes:

- Prior to retinal isolation, keep the fish in the dark (>3 h) to facilitate the separation of the pigmented epithelium from the retina.
- Remove fish from the experiments if any sign of inflammation or bleeding is noticed.
- Pay attention to the total time of dissection not exceeding 30 min, as the activity of the papain will become compromised.

#### 3.3.3. Tissue digestion and cell dissociation – 1 h

1. Add the preheated, activated papain (20 μL) to the pre-digestion solution (980 μL) to obtain an active digestion solution with a final concentration of 16 U/mL papain and 10 U/mL DNase.
2. Filter-sterilize with a 0.22 mm filter and collect in a conical tube (5 mL).
3. Using a cut P1000 tip, transfer the retinas carefully into this active digestion solution and incubate at 30°C for 45 min.
4. Gently flick the suspension during digestion for an even distribution of the papain enzyme.
5. To stop the digestion, move the retinal tissue into a low adhesion vial (1.5 mL) filled with fresh, pre-heated fish culture medium.
6. Rinse 2x with sterile fish medium.
7. Dissociate the retinas mechanically by trituration in a low volume of medium (200 μL). Pipette gently but with a steady pace with a P200 pipette for no more than 20 times to avoid damaging the cells (Grozdanov et al., 2010).

##### Notes:

- To prevent attachment and subsequent loss of cells on the inside of plastic pipette tips, rinse all tips with serum-supplemented fish medium before pipetting cell suspensions.
- Avoid excessive pipetting or the formation of air bubbles during trituration as this will damage the cells and lead to a reduced viability.
- Limit the amount of medium used to transfer retinas into the activated digestion solution, to avoid dilution of the papain enzyme which might result in suboptimal digestion.

#### 3.3.4. Cell loading and culture – 30 min

1. Dilute the dissociated cell sample in 1 mL of fish medium and centrifuge at 30°C and 200 RCF for 5 min to remove dead cells and debris.
2. Carefully discard the supernatants and resuspend the pellet in 400 μL fish culture medium.
3. Count the number of cells and check the viability using an automated cell counter and Trypan blue solution (1:1 v/v). Aim toward a viability of >70%.
4. Remove the fish medium from all wells (but not channels) and plate a volume corresponding to 1.5×10^6^ (150 μL) or 1.95×10^6^ (200 μL) retinal neurons in the SDC of the SND450 or SOC450 device, respectively. Place the pipette tip as close as possible to the opening of the main channel (SND450) or the microgrooves (SOC450) to position as many cells as possible near the microgrooves.
5. Add half of this volume of cell suspension (i.e., 75 and 100 μL for, respectively, the SND450 and SOC450 MFDs) to the wells and channel of the AC. In addition to the Laminin and hydrostatic gradients, these cells will facilitate axonal outgrowth into the AC.
6. Place the MFD in a sterile petri dish and inspect under an inverted microscope for any signs of leakage or air bubbles inside the channels.
7. Incubate for 5 h at 30°C and 5% CO_2_ to let the cells adhere.
8. To facilitate attachment of neurons in the SDC as close as possible to the microgrooves, place the petri dish containing the MFD slightly tilted inside the tissue culture incubator.
9. After 5 h, gently add 100 μL of fresh pre-heated fish medium to the SDC, and 80 μL to the AC, to sustain survival of the densely seeded retinal neurons.
10. The next morning (DIV 1), remove ± half of the medium from the AC (100 μL) and SDC (150 μL).
11. Add 180 μL of fresh preheated CNB medium to the SDC, and thereafter 120 μL CNB to the AC to promote outgrowth and network formation, and reestablish the volumetric gradient from the SDC to the AC.
12. At DIV 1–4, refresh half of the medium every 24 h, as explained for DIV 1 (step 10–11). For cultures kept beyond DIV 4, medium changes can be performed every other day.
13. Monitor the cells daily using an inverted fluorescence microscope to keep track of survival and neurite outgrowth/network formation.

##### Notes:

- Due to the small size of the zebrafish retinal neurons, remaining debris in the cell suspension might occasionally be mistaken for dead cells during automated cell counting. Therefore, always qualitatively confirm the viability obtained by the automated cell counter using an inverted microscope.
- To avoid that the pressure inside a fully loaded AC would limit the numbers of neurons in the SDC adhering close to/growing into the microgrooves, always seed cells first in the SDC, and then in the AC. During medium changes, always firstly remove medium from the AC, then, remove and replace medium in the SDC, and finish by adding fresh medium into the AC.
- As adult zebrafish retinal neurons do not firmly attach to the glass surfaces in a microfluidic setup, perform medium changes gently and very slowly.
- From DIV 3, cells will start to cluster together (as explained in detail in Section 4.1). However, these clusters should be differentiated from aggregates of dead, floating cells, which arise due to improper cell dissociation, inadequate coating or harsh medium changes that can detach the vulnerable retinal neurons.

### 3.4. Axotomy – 15 min (DIV 3)

To study axonal regeneration of adult zebrafish RGCs, a dual vacuum-assisted axotomy from both the top and the bottom well is performed at DIV 3, all as previously described (Taylor et al., 2005; Park et al., 2006; Zhou et al., 2016).

1. First, connect the vacuum aspirator pump to an autoclaved P1000 tip without filter.
2. Turn the vacuum pump on and remove all medium from both AC wells.
3. Position the pipette tip right at the entrance of the channel inside the top well of the AC.
4. Swiftly aspirate all medium inside the AC and stop as soon as the channel is completely emptied.
5. Slowly refill the AC channel with preheated medium.
6. Remove, if needed, again any medium inside the AC wells and perform a second aspiration, this time via the entrance of the channel inside the bottom well of the AC.
7. Lastly, remove half of the medium (150 μL) from the SDC well and add 180 μL fresh, preheated CNB medium to the SDC and 120 μL to the AC, as described above (Section 3.3.4, step 10–11).
8. Evaluate the axotomy using an inverted fluorescence microscope and return the MFDs to the tissue culture incubator.

#### Notes:

- To maximize the opportunities to visualize axonal regeneration, always confirm that a sufficient number of axons, preferably more than 30, are growing into the AC before performing an axotomy. If not enough axons have reached the AC, but many axons are actively growing in the microgrooves, axotomy can be postponed till DIV 4. If the number of axons in the AC is still low by DIV 4, remove the MFD from the experiment.
- Only perform 1 dual aspiration (once from each AC well) and stop each round as soon as all medium is removed from the channel to prevent damage to axon shafts in the microgrooves, or somata in the SDC upon excessive aspiration (Taylor et al., 2005; Park et al., 2006; Dai et al., 2022). In general, if not all axons are axotomized, not cutting all axons (one aspiration less) is preferred to damaging the axon shafts of some axons (one aspiration more), because regenerated axons can always be distinguished from uninjured ones based on confocal time-lapse imaging and overview pictures taken before and upon injury.
- Exceptionally, air bubbles that appear in the AC channel upon addition of medium after axotomy can be aspirated, as they will obstruct the AC channel, thereby limiting the space for regrowing axons.
- Always restore the hydrostatic gradient from the SDC to the AC after axotomy to avoid as much as possible that injured axons in the AC make a U-turn and grow back into the microgrooves.

### 3.5. Time-lapse live microscopy (DIV 2–4)

To visualize dendritic remodeling and/or mitochondrial dynamics upon axonal regeneration in real-time, time-lapse live cell imaging is performed in the microfluidic cultures for up to 10 h during outgrowth at DIV 2, and during regeneration immediately after axotomy at DIV 3 (0 *h post injury*, hpi). All time-lapse images are acquired using an inverted confocal microscope (Zeiss LSM900) equipped with a 20× 0.8NA objective, a camera for epifluorescence imaging and a stage top incubation chamber.

#### 3.5.1. Preparation – 45 min

1. Sterilize the incubation chamber with 70% ethanol.
2. Fill the water bath of the incubation chamber completely with sterile ultrapure water.
3. Open the gas cylinder valves to enable CO_2_ gas to flow into the incubation chamber and set the CO_2_ concentration value to 5%.
4. Fill an assembled MFD with preheated CNB medium and position it inside the incubation chamber. This MFD will serve as an equilibration tool.
5. Place the, sterilized, temperature probe of the incubation system inside one of the AC wells of the equilibration MFD. Ensure that the tip of the probe is completely submerged in medium without touching the glass coverslip.
6. Turn on the incubation main switch and set the imaging chamber temperature to 30°C by appointing following references values to the different heating components: stage heater: 30°C, bath heater: 38°C, top heater: 40°C, CO_2_: 5%.
7. Incubate for 30 min to equilibrate the chamber.
8. Determine the actual temperature of the medium and adjust, if needed, the setting values of the different components to achieve a medium temperature of 30°C. Importantly, always adjust all components proportionally.

##### Notes:

- It is essential to have CO_2_ gas running during all equilibration (and experimental) steps, as the cold incoming gas will affect the temperature inside the chamber.
- Since the actual temperature inside the chamber varies with changing environmental conditions, maintain a stable room temperature, and perform these equilibration steps before each set of experiments.

#### 3.5.2. Axonal outgrowth and regeneration – 10 h

##### 3.5.2.1. Axonal outgrowth (DIV 2)

1. To maximize survival and outgrowth, perform a medium change (as described in Section 3.3.4 step 10–11) right before imaging.
2. Position the MFD inside the incubation chamber and place the temperature probe inside one of the AC wells.
3. Using the confocal Airyscan (Multiplex CO-2Y), acquire a mosaic overview picture of the central part of the MFD, visualizing all microgrooves and a region of ±250 μm of the SDC and ± 500 μm of the AC surrounding them.
4. Based on this overview picture, select a representative region of interest (ROI) where numerous axons can be seen at the border of the SDC and microgrooves, or growing into the microgrooves. Take enough margin in the AC to enable visualization of long outgrowing axons.
5. Acquire time-lapse images with a 20x objective for 5–10 h with a frame interval of 30 min. Use epifluorescence imaging and 4×4 camera binning to maximize speed and avoid phototoxicity, and Definite Focus to prevent focal drift during imaging.
6. After imaging, change medium and return the device to the incubator.

##### 3.5.2.2. Axonal regeneration (DIV 3)

1. Position the MFD inside the incubation chamber and place the temperature probe inside one of the AC wells.
2. Generate an overview picture of the area surrounding the microgrooves as explained above.
3. Inside a biosafety cabinet, perform the axotomy as described in Section 3.4.
4. Immediately transfer the MFD to the incubation chamber of the microscope and acquire an overview picture to evaluate the success of the injury and to identify uninjured neurites.
5. Perform time-lapse imaging directly upon axotomy (DIV 3, 0 hpi) using epifluorescence imaging for 5–10 h with a frame interval of 30 min, all as explained for axonal outgrowth (Section 3.5.2.1).
6. At 24 hpi (DIV 4), create another overview picture to assess overall regeneration and differentiate newly outgrowing axons from actual regenerating ones (see detailed notes).

###### Notes:

- Always minimize exposure to ambient air or lower temperatures to prevent contamination and cell death.
- Handle and transport MFDs with extreme care, to avoid detachment of the cells.
- To avoid photobleaching and maximize survival of imaged RGC neurites, set the power of the excitation lamp and the exposure time to a minimum during each experiment.
- Do not perform medium changes before imaging axonal regeneration at DIV 3, as medium changes are included in the axotomy protocol and unnecessary successive medium changes will disturb the microenvironment of the cells.
- Regularly monitor the sample and check all variables (i.e., temperature, CO_2_, humidity, medium level) before and throughout each experiment. Increased evaporation (due to elevated temperatures, low humidity) or acidification (inappropriate CO_2_ levels) can affect survival and outgrowth as well as axonal transport of the retinal neurons (Padilla and Lyerly, 1986; Bohnert and Schiavo, 2005).
- Of note, in addition to regrowing axons, newly outgrowing axons appear in the AC between axotomy and 24 hpi. Occasionally, these axons do not grow in empty grooves, but rather exploit microgrooves already occupied by regenerating or degenerating axons (Supplementary Movie 1). As a result, they might be misidentified as regenerating axons on overview pictures taken at 24 hpi. To avoid this misinterpretation and exclude these newly outgrowing axons from analyses, always consult time-lapse movies when evaluating (the amount of) regrowth.

#### 3.5.3. Dendritic remodeling – 3 h (DIV 3)

As discussed in more detail in Section 4.1, adult zebrafish retinal cultures require very high densities for survival and outgrowth, which hinders the visualization of individual RGC somata and their dendrites inside the SDC of gap43 microfluidic setups. Therefore, a sparse labeling approach is needed to enable identification of individual neurites and cell bodies from the axons extending into the AC, while still guaranteeing the desired density to promote outgrowth and network formation. Here, a mixed culture of retinal neurons isolated from transgenic reporter (gap43) and WT fish is seeded in the SDC, at a ratio of 1:25 (reporter/WT) using practices described in Section 3.3.4. By performing time-lapse live imaging immediately after axotomy (DIV 3, 0 hpi) in the SDC of these microfluidic models, dendritic changes during RGC axonal regeneration can be visualized.

##### 3.5.3.1. Image acquisition

1. Acquire overview pictures and perform axotomy as described in Section 3.5.2.2.
2. Select an ROI that shows one or a few isolated, axotomized RGCs with segregated dendrite-like neurites in the SDC.
3. As it is known from first observations that dendritic remodeling starts immediately after axotomy, perform time-lapse live imaging as soon as possible after axotomy for 3 h with a frame interval of 10–15 min. Use the confocal Airyscan (Multiplex CO-2Y) to maximize resolution and speed and use the Definite Focus to prevent focal drift during imaging.
4. After imaging, change medium and return the device to the incubator.

##### 3.5.3.2. Anticipated: Image processing and analysis

Dendritic changes can be quantified on confocal still pictures of time-lapse movies using the Simple Neurite Tracer plugin of ImageJ software to perform a Sholl analysis. This analysis allows to measure the complexity of the dendritic arborization by counting the number of intersections between the neurites and concentric circles centered on each individual soma.

#### 3.5.4. Mitochondrial dynamics – 1 h (DIV 2–4)

To investigate intraneuronal mitochondrial motility during axonal outgrowth and regeneration, retinal neurons isolated from gapmito fish, with labeled mitochondria (green) in growing RGCs (red), are seeded in MFDs as described in Section 3.3.4. To obtain a complete overview of the mitochondrial movement during axonal outgrowth and regeneration, time-lapse live imaging is performed in isolated gapmito axons at DIV 2 and DIV 3 (0–5 hpi). Afterwards, kymographs are generated from the resulting time-lapse movies to track single mitochondria and characterize their dynamics (Cartoni et al., 2017; Atkins et al., 2022; Emily et al., 2022).

##### 3.5.4.1. Image acquisition

1. To maximize outgrowth and survival of RGCs, change medium right before imaging.
2. Acquire overview pictures and, when interested in imaging mitochondrial mobility during the regenerative process, perform axotomy as described in Sections 3.5.2 and 3.4, respectively.
3. Based on these overview pictures, select an ROI with one or a few isolated axons and perform time-lapse imaging in the most distal part of these outgrowing (DIV 2) or regenerating (DIV 3) axons in the microgrooves or AC.
4. Use the confocal Airyscan (Multiplex CO-2Y) and acquire images with a 5-s frame interval for up to 10–30 min.
5. After imaging, return the device to the incubator.

##### 3.5.4.2. Image processing

To generate kymographs from the obtained time-lapse movies, open the raw time-lapse imaging data in ImageJ (Wang and Schwarz, 2009; Emily et al., 2022).

1. If needed, adjust brightness and contrast to facilitate visualization of individual mitochondria.
2. Use the Segmented Line Tool in the ImageJ toolbar to manually draw a line from the most proximal (closest to the soma) to the most distal end (at the growth cone) of the selected axon.
3. Create the kymograph by running the Kymograph plugin and KymographBuilder tool.
4. Select the EGFP channel from the resulting images and save for future analyses.
5. Repeat for all axons in one time-lapse movie.

###### Notes:

- RGC axons occasionally grow over one another in the AC, which might complicate the characterization of mitochondrial motility in individual neurites. Always screen for axons that are isolated in the AC or microgrooves and have a clear (proximal to distal) orientation to avoid misinterpretation of anterograde and retrograde movement. Exclude axons that cannot be unambiguously traced.
- As mentioned above (detailed notes in Section 3.5.2.2), newly outgrowing axons appear in the AC (Supplementary Movie 1) in addition to regenerating axons. Consult overview pictures and time-lapse movies for correct interpretation of the axonal identity.
- Axonal growth cones are very dynamic during regeneration (Supplementary Movie 2), which might complicate registration in the distal part of the axon during analysis. Adjust the diameter of the Segmented Line Tool to assure that the full diameter of the axon, including the growth cone, is traced at any point through time.
- Additionally, KymoAnalyzer (an open-source ImageJ package) can be used for semi-automated analysis of various transport parameters (Neumann et al., 2017).
- As an alternative to using retinal cells of the gapmito fish, mitochondria can be labeled using MitoTracker Red FM dye.

1. Hereto, dilute the MitoTracker stock solution (1 mM) to a final working concentration of 0.2 μM in pre-heated CNB.
2. Remove half of the medium in the wells (and channel) of both the SDC (150 μL) and AC (100 μL) and replace for 150 and 100 μL of the dye solution to obtain a final working concentration of 0.1 μM MitoTracker red in, respectively, the SDC and AC.
3. Incubate for 2 h.
4. Wash the cells 2× with preheated CNB medium and return the cells to the incubator for a minimum of 12 and a maximum of 24 h before imaging.
5. Perform imaging as described in Section 3.5.4.1.

##### 3.5.4.3. Anticipated: image analysis

From these kymographs, several parameters can be obtained and analyzed, including the (average) velocity, (average) direction, (average) moving frequency, moving distance and the number of motile and stationary mitochondria. Comparing these data between the different conditions will enable to quantify how axonal mitochondrial motility changes throughout the regenerative process (Basu et al., 2020).

##### 3.5.4.4. Anticipated: somatodendritic mitochondrial dynamics

By performing time-lapse imaging for mitochondrial motility (as described in Section 3.5.4) in mixed retinal cultures (made as described in Section 3.5.3), changes in mitochondrial dynamics can also be visualized in the dendrite-like neurites of the isolated RGCs. As such, dendritic remodeling and mitochondrial mobility can be studied concurrently during axonal regeneration at DIV 3.

### 3.6. Endpoint immunostainings – 2 days (DIV 2–4)

Time-lapse live cell imaging experiments can be complemented with endpoint immunofluorescent stainings on fixed cells. For example, immunostaining for Map2 and Tau can be performed to identify RGC axons and dendrites. In addition, performing a nuclear counterstain with DAPI allows to evaluate the overall density of the cultures.

#### 3.6.1. Fixation

1. Carefully remove most of the medium from the SDC and AC wells (but not channels) and replace with a filtered 4% PFA in PBS solution using practices explained in detail in Section 3.2.2.
2. Incubate for 10 min.
3. Rinse the fixed cells 3× for 10 min with filtered PBS.
4. Store in the dark at 4°C for up to 1 week or proceed directly with immunocytochemistry.

##### Notes:

- Although fixation should not be performed in a sterile environment, prior filtration of all solutions will minimize contamination of the device with debris or dirt that might interfere with imaging.
- Do not remove medium from the channels and ensure that the cells are always covered by a small amount of liquid during any of the fixation and/or staining steps to avoid disruption of the fragile neuronal networks.
- Assure an adequate incubation time (>10 min) and volume difference between the SDC and AC (>50 μL) for all fixation, washing and staining steps (Section 3.6.2) to guarantee that full length of the microgrooves is treated/stained.

#### 3.6.2. Immunocytochemistry

1. First, rinse both SDC and AC with PBST for 10 min.
2. Perform a 1-h blocking step with BSA diluted 1:100 in PBST to block non-specific binding sites for the primary antibody. Use similar practices as mentioned in Section 3.2.2.
3. Incubate the MFDs overnight with the primary antibodies (mouse anti-Map2, mouse anti-Tau) diluted 1:200 in PBST.
4. Rinse 3× with PBST for 10 min.
5. Incubate the cells with secondary antibody (DAM-Alexa 647) diluted 1:300 in PBST for 2 h.
6. Was 3× with PBST for 10 min.
7. Counterstain the nuclei for 30 min using DAPI.
8. Rinse 1× with PBS.
9. To preserve fluorescence, store in the dark at 4°C until imaging (maximally 2 weeks).

##### Notes:

- Execute all steps while gently shaking at room temperature in the dark.
- Place the MFDs in a humidified chamber during long (>30 min) incubation steps to prevent evaporation of fluid from the small wells.

## 4. Representative and anticipated results

### 4.1. Generation of an adult zebrafish retinal cell culture protocol

**Figure 2:**
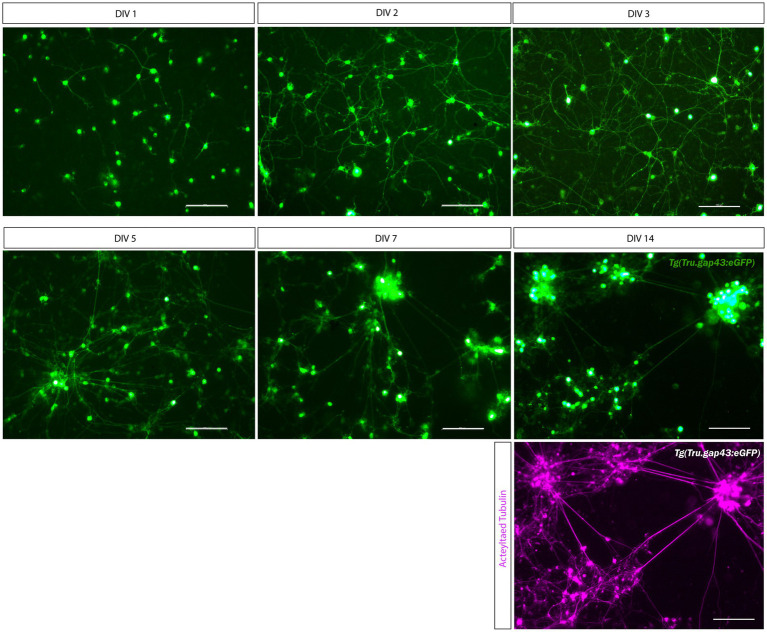
Long-term primary cell culture of adult zebrafish retinal neurons at DIV 1–14. Adult zebrafish retinal neurons can successfully be cultured using our newly established protocol. Live cell images taken at DIV 1–14 in a *Tg(Tru.gap43:GFP)^mil1^*(gap43) retinal cell culture demonstrate that GFP-expressing RGCs start to sprout neurites at DIV 1, and extensive networks are established from DIV 3 onwards. At DIV 5, clusters of retinal neurons start to form that increase in size during the following days in culture, as more and more neurons crowd together. As a result, cultures appear less evenly spread throughout the well. Time-lapse imaging discloses that these clusters are formed by active migration of retinal neurons (Supplementary Movie 2). Although neurons survive up to DIV 14, almost all neurons have grouped together at this point, and GFP expression is strongly decreased in RGC neurites. Immunostainings for the neurite marker Acetylated tubulin (magenta labeling, bottom panel) reveal healthy neurons with extensive neurites at DIV 14, indicating that the observed decrease in GFP labeling is due to the downregulation of the *gap43* promoter, rather than dying of the cells. Scale bars: 100 μm. DIV, days *in vitro*; ONC, optic nerve crush; RGC, retinal ganglion cell.

The first step in establishing a new microfluidic model was the creation of a successful adult zebrafish retinal culture as initial attempts, using published protocols for zebrafish retinal neurons and/or explants, resulted in a high mortality and an almost complete absence of neuronal outgrowth (Schwartz and Agranoff, 1981; Grozdanov et al., 2010; Chen et al., 2013). Therefore, different parameters within the experimental design, including culture medium, plate coating and cell seeding density, were individually optimized until a successful protocol was established that enabled the culture and maintenance of outgrowing adult zebrafish retinal neurons in 96-well plates (Supplementary Figure S2; Figure 2).

In summary, an ONC is performed in adult zebrafish 2 days prior to retinal isolation and cell seeding (DIV-2), and used as a conditioning lesion (CL) to prime RGCs for regeneration. A CL is known to accelerate axonal regrowth in both the PNS and CNS of mammals and teleost fish, and to stimulate outgrowth upon culturing (Landreth and Agranoff, 1976; Schwartz and Agranoff, 1981; Becker and Becker, 2002; Schwartz, 2004; Elsaeidi et al., 2014; van Niekerk et al., 2022). In line with these findings, also in our model, a CL indeed promotes *in vitro* neurite outgrowth and network formation compared to non-conditioned cultures (Supplementary Figure S2A; Table 1). Two days later (DIV 0), retinal neurons are isolated and seeded on a PLL-and Laminin-coated surface at a density of 22,000 cells/mm^2^ and incubated at 30°C and 5% CO_2_, all as described in detail in Section 3.3.4 (Supplementary Figures S2B–D).

**Table 1:** List of key practices to sustain a successful adult zebrafish retinal cell culture in SOC450 MFDs.

Approach	Reasoning
ONC as an *in vivo* conditioning lesioning to prime *in vitro* RGC axonal outgrowth.	Outgrowth and network formation is more limited and delayed in non-conditioned uncrushed RGCs.
Consecutive overnight PLL and Laminin coating.	PLL coating results in an increased attachment, as well as reduced cell clustering, while Laminin improves outgrowth, all as compared to solely PLL or Laminin or uncoated surfaces.
High seeding density of 22,000 cells/mm^2^.	Higher seeding densities facilitate cellular interactions and connections and result in augmented RGC survival and neurite outgrowth.
Seeding cells in fish medium at DIV 0 and transitioning to CNB medium from DIV 1 onwards.	Serum-containing fish medium sustains glial cells, which are vital to maintain neuronal health. Serum-free CNB medium prevents glial overgrowth and facilitates neuronal outgrowth due to the B27 supplementation.
Optimal culture duration of 4 days (DIV 0–4) with execution of experimental procedures between DIV 2–4.	Axonal outgrowth is a very fast process in cultured adult zebrafish RGCs. *gap43*-driven expression in dissected and cultured RGCs starts to decrease from DIV 5 onwards, thereby limiting investigations from this point on.
Addition of conditioned target cells in the AC.	Primed RGCs added to the AC produce growth factors that attract axons of RGCs outgrowing from the SDC.
Medium changes every day (DIV 0–4) or every other day (DIV 5–14) by replacing half of the medium with fresh medium.	The small size of zebrafish RGCs, in combination with the requirement for a high seeding density, requires daily medium changes to sustain neuronal survival and outgrowth at the start of the culture. From DIV 5 onwards, medium changes can be performed every other day.

The high seeding density is critical to support outgrowth and survival of the cells, as demonstrated by an augmented network formation and higher number of outgrowing/surviving neurons with increasing seeding densities (Supplementary Figure S2D). Moreover, optimal neuronal outgrowth and network development are achieved when fish medium is used for cell seeding at DIV 0 and replaced by CNB medium for culturing from DIV 1 onwards (Supplementary Figure S2C). This is likely because serum-supplemented fish medium first sustains glial cells *in vitro*, which are known to be important for neuronal survival and growth (Garcia et al., 2002). Switching to serum-free CNB medium thereafter prevents glia from proliferating and taking over the culture, and provides additional support for neuronal outgrowth due to the B27 supplement.

Upon culturing of adult *Tg (Tru.gap43:GFP)^mil1^* (gap43) retinal neurons with this optimized protocol, GFP expression can already be detected in RGC somata 5 h after seeding as a result of a CL at DIV-2. Indeed, upon ONC injury, the *gap43* promoter, and thereby GFP expression, is activated in RGCs specifically (Udvadia, 2008; Diekmann et al., 2015). GFP-expressing RGC neurites start to sprout at DIV 1, and clear network formation is observed from DIV 3, all without the need for growth factor supplementation. Immunolabeling for the neuronal marker Acetylated-tubulin reveals that healthy cultures can be maintained for up to 14 days, but at this time point, GFP expression is strongly decreased, and excessive cell clustering hampers the identification of individual neurons (Figure 2). Indeed, cultured adult zebrafish retinal neurons actively migrate toward one another, which results in the formation of cell clusters (Supplementary Movie 3). These clusters appear from DIV 3 and increase in size and complexity during the following days (Figure 2). As clustering seems to support survival and neurite outgrowth, high cell densities might be favored because they facilitate interneuronal contacts. Altogether, this protocol enables to maintain a long-term culture of primary adult zebrafish retinal neurons with extensive neurite outgrowth and network formation.

### 4.2. Optimization of an adult zebrafish retinal microfluidic model

**Figure 3:**
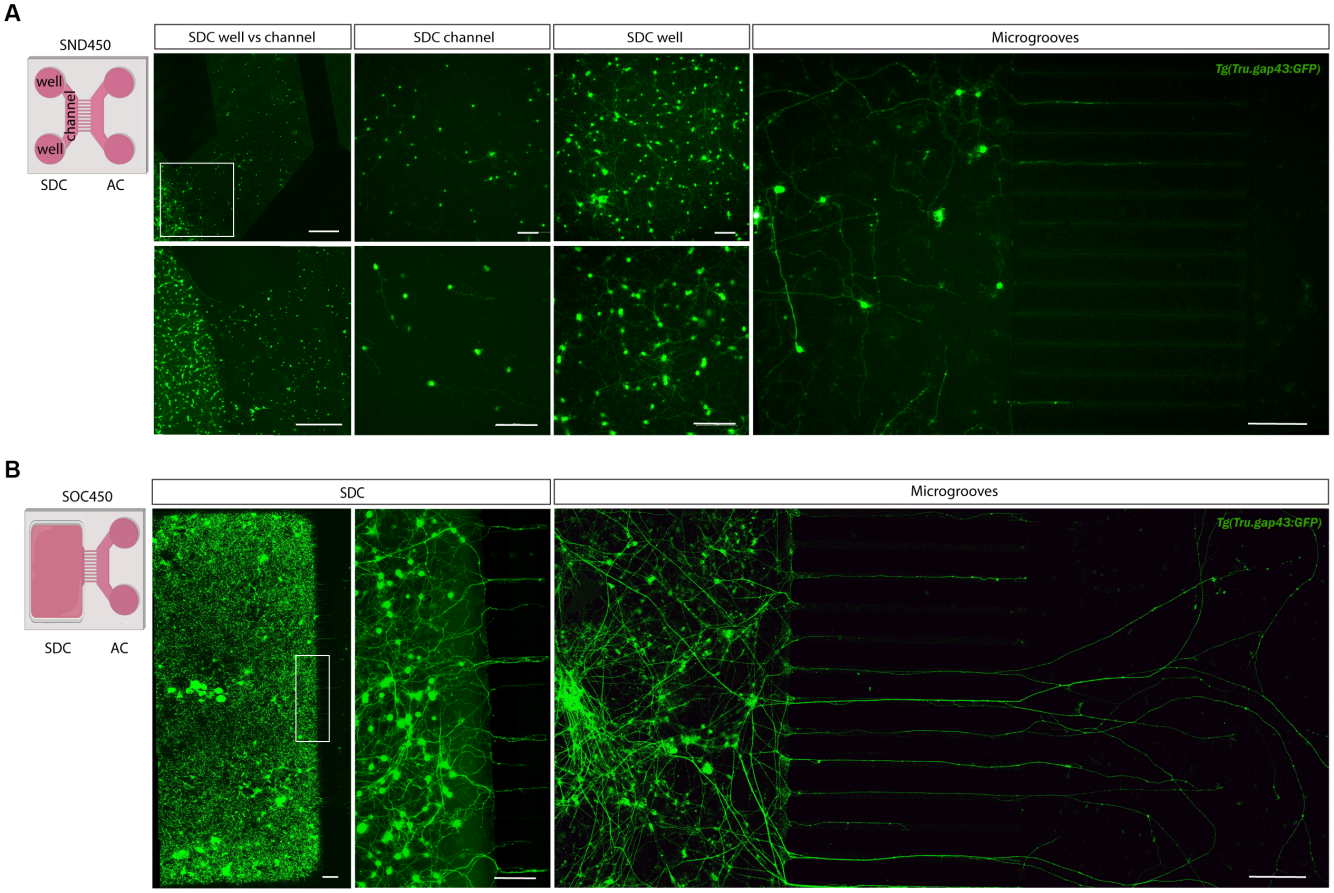
Adult zebrafish retinal cell culture in a microfluidic setup at DIV 3. **(A)** Adult zebrafish retinal neurons cultured in SDN450 MFDs, display limited outgrowth and network formation. Live cell images taken at DIV 3 demonstrate that *Tg(Tru.gap43:GFP)^mil1^* (gap43) retinal neurons, loaded in the wells and channel, do not attach properly into the channel, which causes a flow of neurons from the channel toward the wells after seeding (DIV 0) or addition of medium (DIV 0–3). As a result of this low density in the SDC channel, neurons display strongly reduced neurite outgrowth and network formation compared to neurons growing in the SDC wells, and only a few neurites grow into the microgrooves at DIV 3. **(B)** In contrast, at DIV 3, gap43 retinal neurons seeded in the SDC of an open compartment SOC450 MFD form an extensive neuronal network throughout the entire SDC, including the areas bordering the microgrooves. Consequently, spontaneous RGC outgrowth into the microgrooves is achieved in this setup, with numerous axons growing far into the AC at DIV 3. Scale bars: 500 μm (overview pictures), 100 μm (detailed pictures). AC, axonal compartment; CNB, complete neurobasal medium; DIV, days *in vitro*; MFD, microfluidic device; RGC, retinal ganglion cell; SDC, somatodendritic compartment.

In a next step, the optimized protocol was implemented in a commercially available microfluidic setup. For the initial trials, we used SND450 MFDs, where both the SDC and AC consist of two wells connected by a channel, that were assembled and coated with PLL and Laminin at DIV-2 (Figure 3A). After dissociation and isolation, all performed as described above, retinal neurons were seeded in the SDC wells and channel at DIV 0. Unfortunately, retinal neurons did not attach properly in the channel of the SND450 MFD and a flow of cells from the channel toward the wells was observed immediately after seeding and upon medium changes in the following days. As a result, cell densities in the channel were too low to support RGC survival and axonal outgrowth into the microgrooves. In contrast, a widespread neuronal network was formed inside the SDC wells from DIV 2 onwards. Despite various attempts to enhance cell adhesion, survival and outgrowth in the SDC channel, only limited improvements were made (for details, see Table 2). Noteworthy, an increase in outgrowth of SDC RGCs axons toward the microgrooves was achieved by adding conditioned retinal cells into the AC. Most likely these cells secrete factors that attract RGC axons (Supplementary Figures S3A,B). However, still only a few axons eventually reached the AC (Figure 3A).

**Table 2:** List of various parameters evaluated to improve RGC adherence, survival and axonal outgrowth in SND450 MFDs.

Approach	Effect	
	Adherence and subsequent survival	Axonal outgrowth
Sonication/autoclavation of both cover slips and PDMS top part.	**−**	
Performing overnight coating for both PLL and Laminin at 37°C instead of 4°C.	**−**	
Providing Laminin coating in the SDC channels but not in the SDC wells.	**−**	**−**
Seeding cells only in SDC channel and not in the SDC wells.	**−**	**−**
Seeding of cells in two steps (with 5 h interval) to increase the total number of cells in the channel.	**−**	**−**
Pre-incubation of the SDC wells and channel with medium, and seeding a small volume of a very concentrated cell suspension in the SDC channel to reduce movement of cells upon seeding.	**+**	**+**
Providing a physical barrier at the border of the wells/channel using Matrigel to prevent movement of cells from the channel into the wells upon seeding.	**−**	**−**
Applying a Laminin concentration gradient from the AC to the SDC.	**−**	**+**
Increasing the volume difference from the SDC to the AC to create a higher hydrostatic pressure toward the AC.	**−**	**−**
Addition conditioned cells to (the wells and channel of) the AC.		**++**

**–,** No improvement; **+**, Small improvement; **++**, Large improvement.

**Figure 4:**
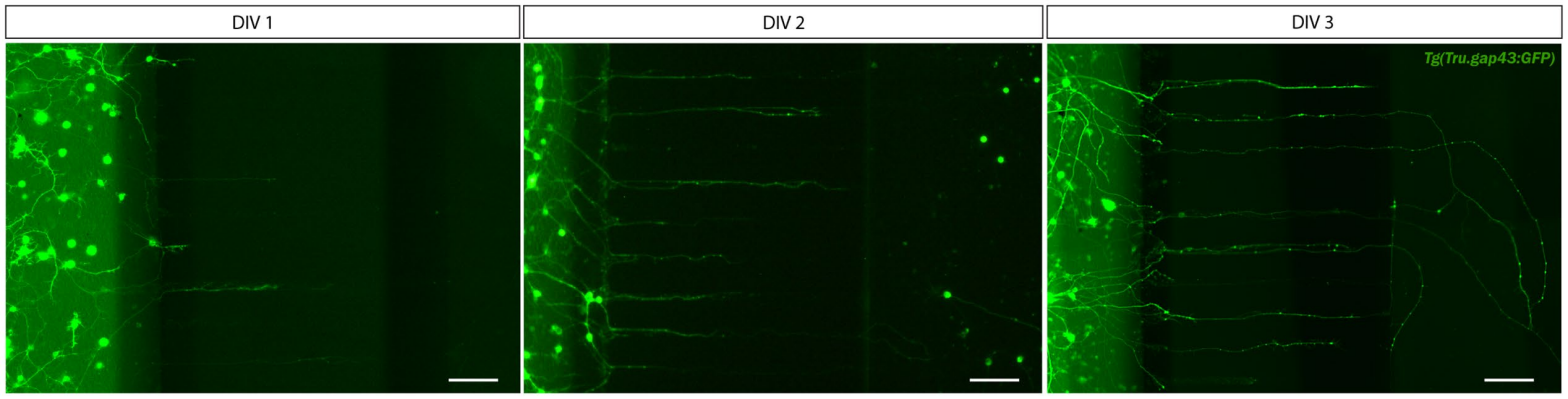
Outgrowth of adult zebrafish RGC axons at DIV 1–3 in a microfluidic setup. Adult zebrafish retinal neurons can successfully be cultured in an open (SOC450) MFD. Confocal live cell images indicate that adult *Tg(Tru.gap43:GFP)^mil1^*(gap43) zebrafish RGCs show early outgrowth and network formation from DIV 1 onwards, and that RGC axons are growing into the microgrooves and toward the AC at DIV 2. By DIV 3, numerous axons have reached to AC or are actively growing into the AC. Scale bars: 100 μm. AC, axonal compartment; CNB, complete neurobasal medium; DIV, *days in vitro*; MFD, microfluidic device; RGC, retinal ganglion cell; SDC, somatodendritic compartment.

Due to the difficulties encountered with the SND450 MFDs, we switched to the more recently developed, open compartment SOC450 MFD (Figure 3B). In this design, the SDC wells and channel are fused into one open compartment, thereby limiting cell movement upon addition/changes of medium. Indeed, when adult zebrafish retinal neurons are cultured in this setup using the same protocol as described earlier, a high and evenly distributed cell density can be maintained throughout the entire SDC. As a result of the increased density near the microgrooves, numerous RGC neurites can be observed growing into the microgrooves from 2 DIV onwards, and by DIV 3, many of these axons have reached the AC (Figures 3B, 4). Due to this immense improvement in outgrowth into the AC, all following steps are only performed using SOC450 MFDs. Importantly, also in this setup, conditioned retinal cells can be seeded in the AC to promote outgrowth toward the microgrooves and AC (Supplementary Figures S3C,D).

### 4.3. Evaluation of axonal outgrowth and regeneration in adult zebrafish RGCs

#### 4.3.1. Vacuum-assisted RGC axonal injury

**Figure 5:**
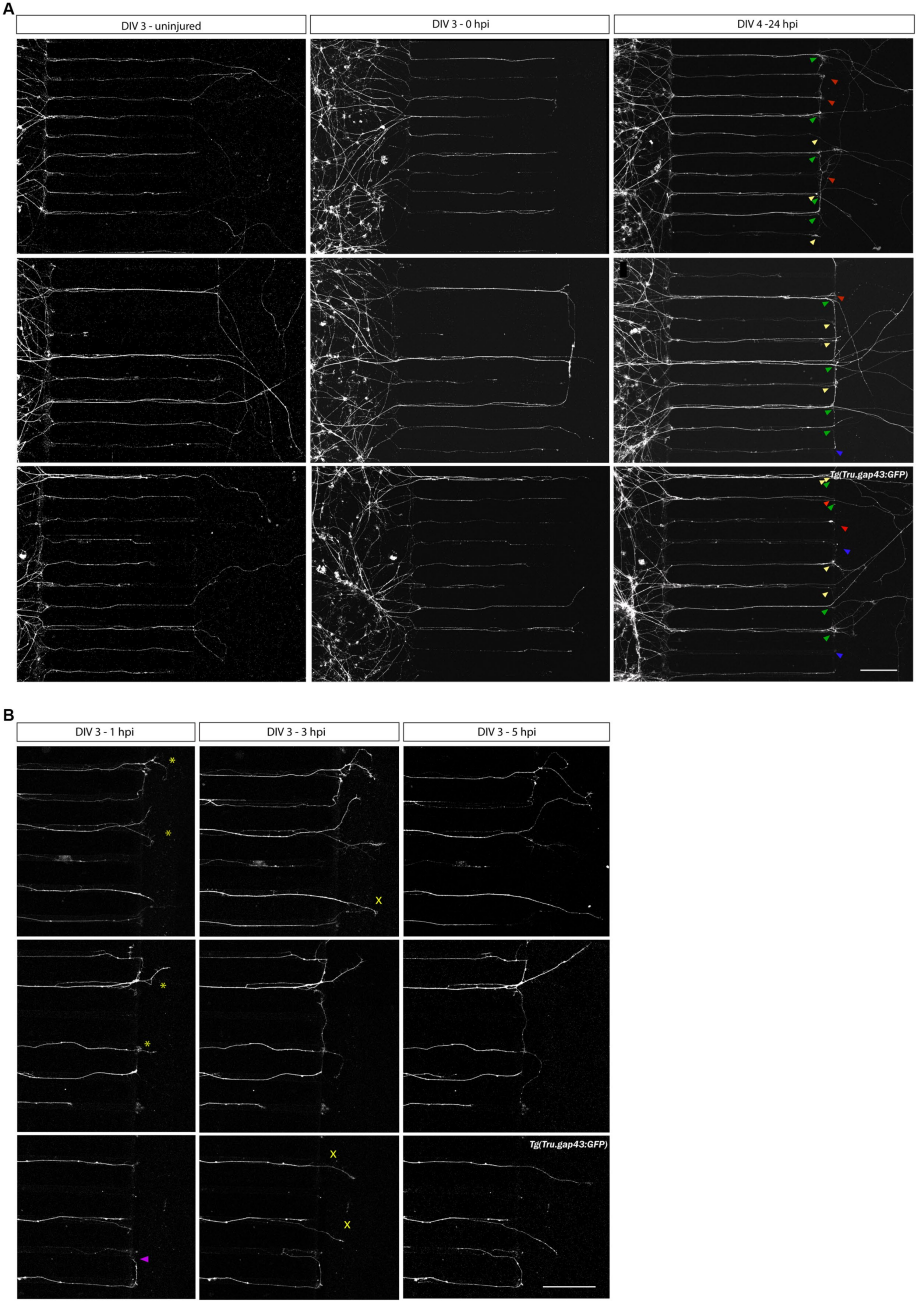
Axotomy and regeneration of adult zebrafish RGCs at DIV 3–4. Representative confocal live cell microscopy images taken before, upon and after injury in the same section of the AC, disclose that adult zebrafish RGCs regenerate spontaneously upon *in vitro* axotomy in SOC450 MFDs. **(A)** At DIV3, when numerous adult *Tg(Tru.gap43:GFP)^mil1^* (gap43) zebrafish RGC axons are growing in the AC (left panels), an axotomy is performed using dual aspiration of all medium in the AC (DIV 3, 0 hpi, middle panels). One day later, at DIV 4 (24 hpi), numerous regenerating axons can be observed in the AC (regenerating axons, green arrowheads in right panels). However, some axons do not regenerate and stay stationary after axotomy (static axons, red arrowheads) or even degenerate upon injury (degenerating axons, blue arrowheads). Lastly, some axons, that did not reach the AC at the time of axotomy, further extend and form newly outgrowing axons that reach the AC in the hours following axotomy (0 hpi-24 hpi) (newly outgrowing axons, yellow arrowheads). These different states of axons can be distinguished using overview pictures at DIV 3 and DIV 4 in combination with time-lapse live imaging (Supplementary Movies 1, 5). **(B)** Representative live cell images taken at different time points after axotomy in the same section of the AC of a gap43 retinal culture after axotomy at DIV 3 reveal that regeneration is a dynamic process, and different axons extend at different moments and with different growth rates. Some pioneering axons start to regenerate immediately upon injury (0–1 hpi, *), while others only emerge after a few hours in the AC (1–3 hpi, ^×^). Of note, occasionally axons make a U-turn and grow back into the microgrooves, rather than regrowing into the AC (purple arrowhead). Scale bar: 100 μm. AC, axonal compartment; DIV, *days in vitro*; hpi, *hours post injury*; MFD, microfluidic device; RGC, retinal ganglion cell.

To study axonal regeneration, a vacuum-assisted axotomy is performed at DIV 3. A successful injury is recognized by a clean cut at the border between the microgrooves and the channel of the AC, while the axonal shaft in the microgrooves as well as the somata in the SDC remain unharmed. Confocal overview images acquired before (DIV 3, uninjured), upon (DIV 3, 0 hpi) and 1 day after injury (DIV 4, 24 hpi), show that axotomized RGC axons display robust and long-range regrowth within 1 day (Figure 5A, green arrowheads). These results indicate that adult zebrafish RGC axons regrow spontaneously in an *in vitro* setup without the need for supplemented neurotrophic factors, unlike most mammalian models of induced regeneration. Notably, these images disclose that at 24 hpi (DIV 4), in addition to regenerating axons (injured and regenerating, green arrowheads, ± 46% of all axons in the AC) also degenerating (injured but undergoing Wallerian-like degeneration, blue arrowheads, ± 8% of all axons in the AC), static (injured but neither regenerating nor degenerating, red arrowheads, ± 15% of all axons in the AC), and newly outgrowing (uninjured, actively outgrowing, yellow arrowheads, ± 33% of all axons in the AC) axons can be observed in/at the border of the AC (Figure 5A). ‘Newly outgrowing axons’ are defined as axons that emerge from uninjured RGCs in the SDC and reach the AC in between the moment of axotomy (0 hpi) and 24 post injury (24 hpi). These different types of axons can be differentiated on overview pictures taken before, upon and after injury when combined with detailed evaluations on time-lapse movies to track each individual axon over time.

Sporadically, regenerating RGC axons turn and grow back into the microgrooves after axotomy, instead of directing straight into the AC (Figure 5B, purple arrowhead). Most often, this U-turning occurs upon inadequate or slow aspiration, whereby axons are pushed to the side of the AC rather than being fully cut. To minimize this phenomenon, a dual aspiration axotomy should be performed quickly and with constant suction power.

#### 4.3.2. Characterization of RGC axonal outgrowth and regeneration using live cell imaging

The RGC axonal outgrowth and regeneration processes can be monitored over time by performing confocal time-lapse live imaging in the microfluidic cultures. Hereto, we first optimized the imaging settings and timing to visualize axonal outgrowth in real-time in adult zebrafish RGCs (Section 3.5.2). The resulting confocal time-lapse movies illustrate that axonal outgrowth is maximally ongoing at DIV 2, with numerous axons actively growing into the microgrooves and AC, and some exceptionally fast axons even growing more than 1,000 μm over an 8-h period (Supplementary Movie 4). After axotomy at DIV 3, axons start to regenerate at different time points after injury, with some sprouting before 1 hpi, while others only start to regrow within 1–3 hpi (Figure 5B:*,^x,^ respectively; Supplementary Movie 5).

**Figure 6:**
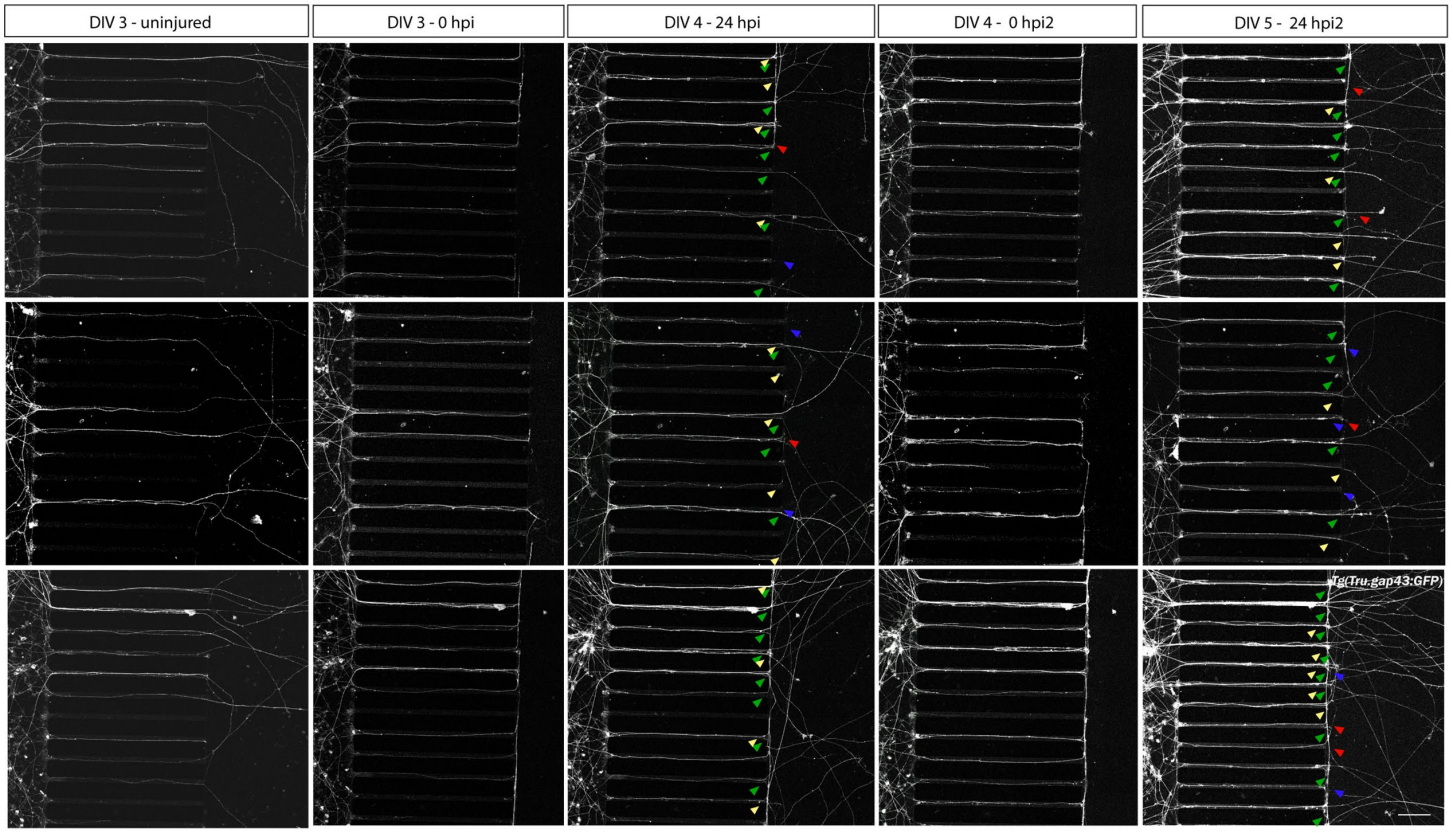
Axotomy and regeneration of repeatedly injured adult zebrafish RGCs at DIV 3–5. Adult zebrafish RGC axons that have successfully sprouted after a first *in vitro* axotomy, regenerate again when a second *in vitro* axotomy is executed. Representative confocal live cell microscopy images taken before, upon and after injury in the same section of the AC of cultured *Tg(Tru.gap43:GFP)^mil1^*(gap43) retinal neurons in an open compartment (SOC450) MFD disclose that 24 h after both the first (DIV 4, 24 hpi, 3th panels) and the second axotomy (DIV 5, 24 hpi2, 5th panels), regenerating (green arrowheads) as well as newly outgrowing (yellow arrowheads), static (red arrowheads) and degenerating axons (blue arrowheads) can be observed in the AC. These results indicate that repeated injuries (*in vivo* ONC, isolation, *in vitro* axotomy) do not negatively impact the spontaneous regenerative potential of cultured zebrafish RGCs. Of note, the high number of outgrowing and regenerating axons at DIV 5 complicates the correct identification of individual axons in the AC. Therefore, no further analyses should be performed on these cultures. Scale bar: 100 μm. AC, axonal compartment; DIV, *days in vitro*; hpi, *hours post injury*; MFD, microfluidic device; RGC, retinal ganglion cell.

Within this optimized adult neuron regeneration model, RGC axons undergo multiple injuries (i.e., *in vivo* ONC, dissociation, *in vitro* axotomy). To refute a potential impact of repeated injuries on the *in vitro* regenerative potential of zebrafish RGC axons, we also performed a second axotomy on already regenerated RGC axons (24 hpi, DIV 4). Confocal still overview pictures taken 1 day after the second injury (24 hpi2, DIV 5), demonstrate that in addition to a few degenerating and static axons, many regenerating and again newly outgrowing axons emerge in the AC, indicating that repeated axotomy does not preclude axonal regeneration (Figure 6, arrowheads).

Notably, we also investigated the outgrowth and regenerative potential of non-conditioned retinal neurons (i.e., non-crushed) in MFDs, and compared them with conditioned cultures harvested from the same fish (left and right eye, respectively). Non-conditioned RGCs only start to sprout at DIV 3, and some axons reach the AC by DIV 4–5, illustrating how their outgrowth is delayed (with ±2 days) and axon numbers reduced (i.e., ± 60% less axons in the AC), as compared to conditioned cultures (Supplementary Figure S4A; Figure 5). Upon axotomy at DIV 4, regenerating [green arrowheads, (± 35% of all axons), as well as newly outgrowing (yellow arrowheads, ± 25% of all axons)] degenerating (blue arrowheads, ± 18% of all axons) and static (red arrowheads, ± 25% of all axons) axons can be observed in the AC at 24 hpi (DIV 5). Again, we observed a notable reduction in both the length and the total number of axons entering the AC at 24 hpi (±75% less axons in the AC) compared to conditioned cultures at 24 hpi (DIV 3) (Supplementary Figure S4B; Figure 5). These findings again confirm our previous observations (Supplementary Figure S2A) and highlight that an *in vivo* CL promotes both neurite outgrowth and injury-induced regeneration *in vitro*. Similar observations have been made for goldfish and zebrafish (explant) cultures, where after 2 weeks in culture, neurite outgrowth could be observed in both conditioned and non-conditioned explants, but neurites remained remarkably shorter in the latter condition (Landreth and Agranoff, 1976; Schwartz and Agranoff, 1981).

### 4.4. Investigation of dendritic remodeling in regenerating adult zebrafish RGCs

#### 4.4.1. Visualization of dendrites of individual regenerating adult zebrafish RGCs

**Figure 7:**
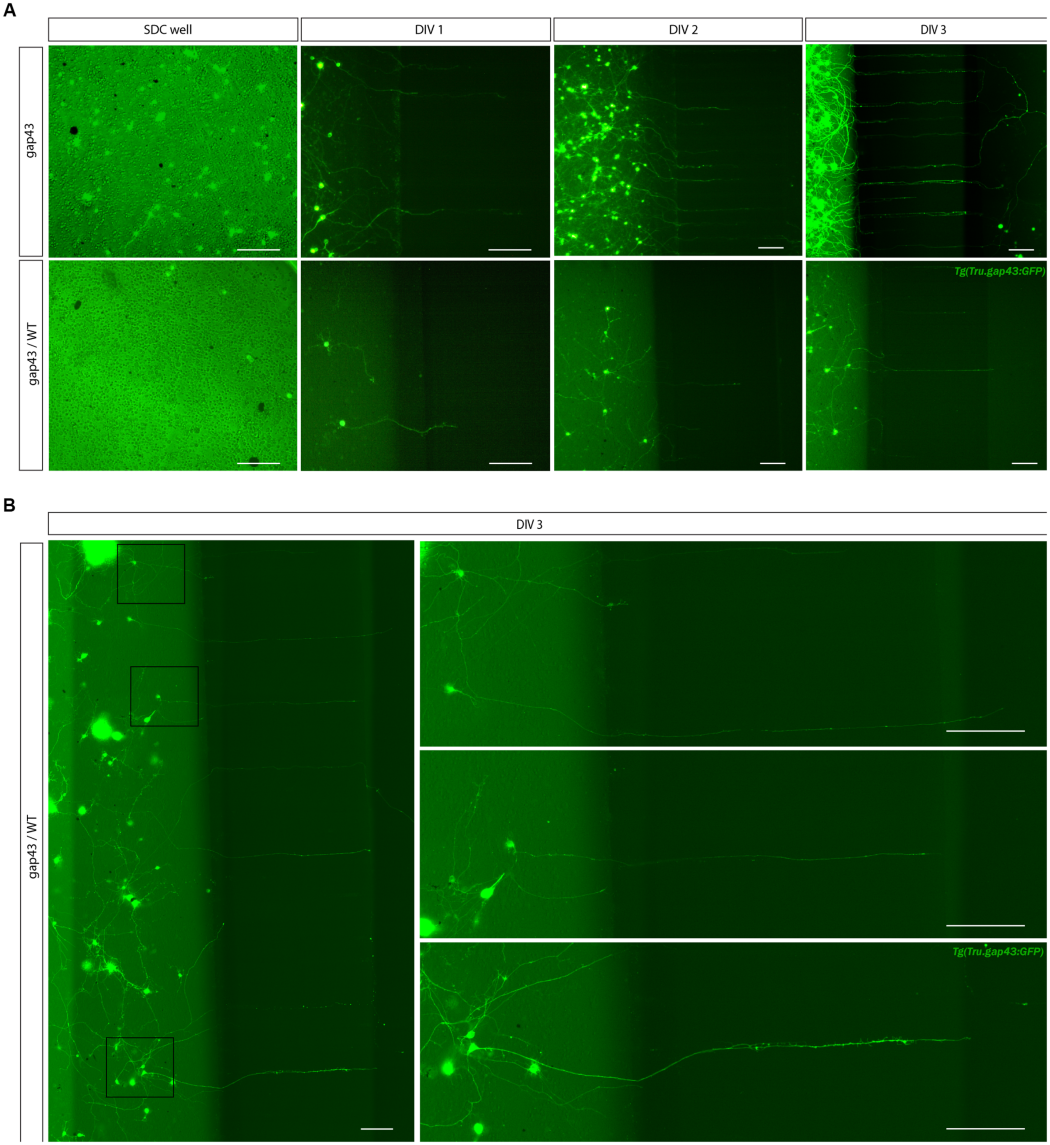
Creation of a sparsely labeled mixed culture to visualize individual adult zebrafish RGCs. To enable characterization of dendritic remodeling during axonal outgrowth, a sparsely labeled, mixed zebrafish retinal cell culture is created. **(A)** Comparison of control *Tg(Tru.gap43:GFP)^mil^* (gap43) and mixed gap43/wild type (gap43/WT) cultures at DIV 1 illustrate that seeding a mixture of cells at a ratio of 1:25 (gap43/WT) in the SDC of a SOC450 MFD results in a more sparsely labeled culture, without compromising cell density, which is essential to sustain outgrowth and network formation. Although most certainly a similar number of axons grow out in both conditions, in the mixed retinal cultures only a few labeled adult zebrafish RGCs become apparent at DIV 1 and can be seen growing into the microgrooves at DIV 2–3. **(B)** Confocal live imaging at DIV 3 demonstrates that in the sparsely labeled mixed cultures, individual RGCs, with axons growing into the microgrooves and AC, can be distinguished in the SDC. Scale bars: 100 μm. AC, axonal compartment; DIV, *days in vitro*; hpi, *hours post injury*; MFD, microfluidic device; RGC, retinal ganglion cell.

The higher density requirement of adult zebrafish retinal neurons for survival and outgrowth (Section 4.1) complicates the identification of individual RGCs and their dendritic trees in the SDC of the MFDs (Figure 7A). To overcome this hurdle, a sparsely labeled culture is achieved through dilution, i.e., by seeding a mixture of cells harvested from labeled gap43 and unlabeled WT retinas in the SDC. This setup allows to visualize both short dendrite-like neurites and axons of individual RGCs while still ensuring a high density. At a ratio of 1:25 (i.e., 78000 gap43 and 1872000 WT retinal cells seeded in the SOC450 SDC) individual labeled neurons do not have overlapping dendrite-like neurites in the SDC, yet enough labeled axons grow out, and regenerate, into the AC (Figure 7B).

#### 4.4.2. Anticipated result: characterization of dendritic remodeling upon axonal injury

**Figure 8:**
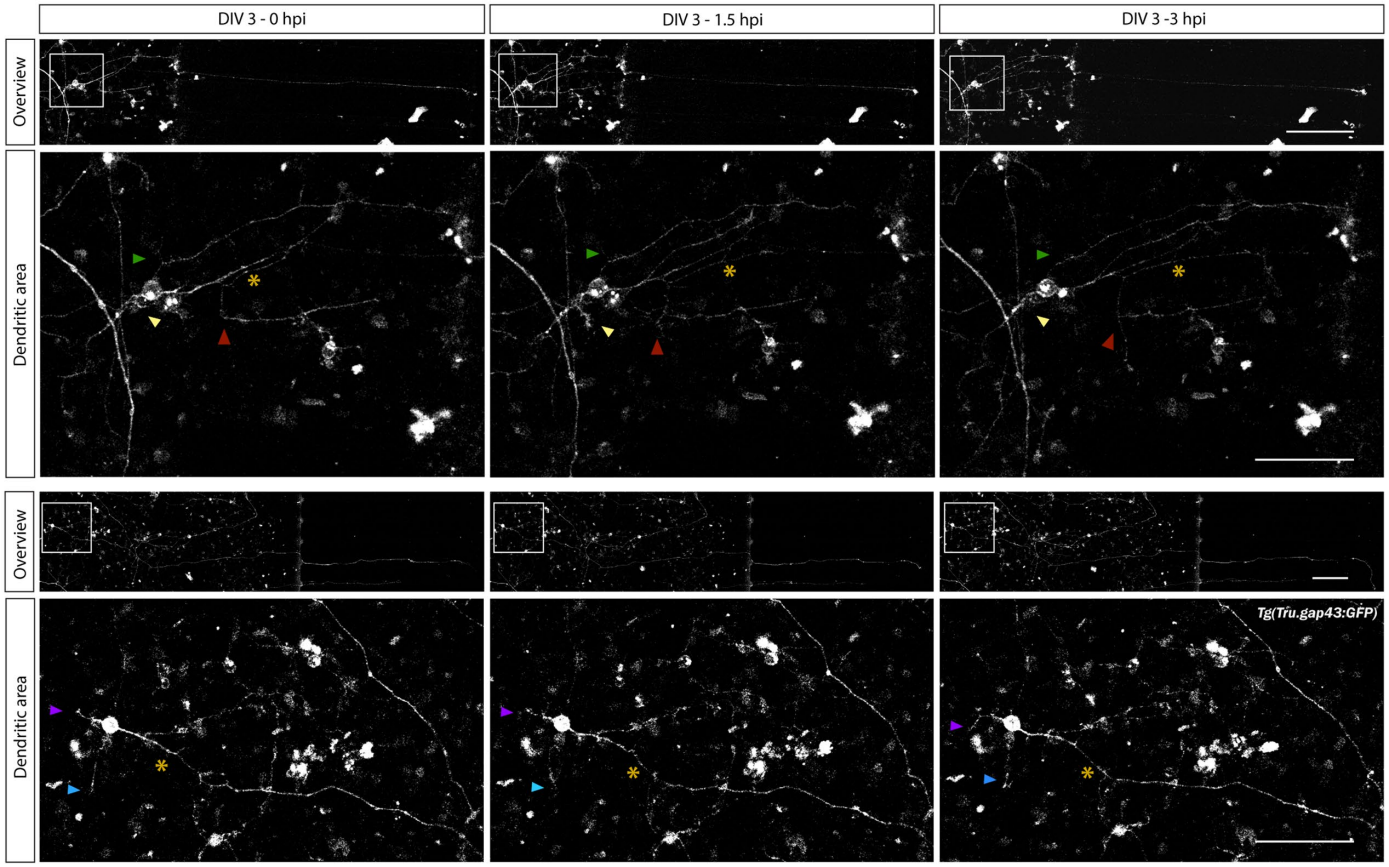
Characterization of changes in dendrite-like neurites during adult zebrafish RGC axonal regeneration. Time-lapse live imaging in sparesely labeled, mixed adult zebrafish retinal cultures seeded in a SOC450 MFD enables characterization of dendritic remodeling during axonal regeneration of adult zebrafish RGCs. Representative confocal still overview pictures of isolated gap43 RGCs (top panels) and detailed images of the dendritic area (bottom panels, with colored arrowheads indicating the same dendrite-like neurites over time and * indicating the axotomized axon) in a mixed *Tg(Tru.gap43:GFP)^mil1^/*wild type (gap43/WT) retinal cell culture illustrate both axonal regeneration and temporary dendritic remodeling (in length and relative position) after axotomy at DIV 3 (0–3 hpi). These first evaluations indicate the potential of this sparsely-labeled setup to visualize and characterize dendritic changes during axonal regeneration of adult zebrafish RGCs. Scale bars: 100 μm (overview pictures); 50 μm (magnified inserts). AC, axonal compartment; DIV, *days in vitro*; MFD, microfluidic device; RGC, retinal ganglion cell; SDC, somatodendrtic compartment.

To characterize dendritic remodeling upon axotomy in these mixed microfluidic retinal cultures, time-lapse live imaging is performed as described in Section 3.5.3. As dendritic shrinkage is reported to occur prior to axonal regrowth of injured zebrafish RGCs (Beckers et al., 2019), and RGC axons start to regrow within 3 h after injury (Section 4.3.2), images are acquired within this time frame. Time-lapse movies taken during a pilot study demonstrate that individual RGCs in the SDC can be monitored during axonal regeneration (Supplementary Movie 6). Moreover, some first qualitative evaluations of these time-lapse movies reveal temporary changes in the dendrite-like neurite area before and during axonal regeneration (Figure 8, arrowheads).

Future quantitative analyses on a larger number of time-lapse experiments will enable to fully characterize the extent of this neurite remodeling, e.g., by performing a Sholl analysis (Ferreira et al., 2014; Bartelt-Kirbach et al., 2016). Here, we provide a first proof of concept that illustrates the application potential of this technique for future analyses.

One limitation within this model is that in our hands, dendrites and axons cannot be identified in cultured adult zebrafish retinal neurons using immunostainings for commonly used dendritic (Map2) or axonal (Tau) markers. Despite multiple trials using different protocols and antibodies, at best only limited Map2 labeling could be achieved in long-term (DIV 14) retinal cultures (Supplementary Figure S5A, arrowhead). Notably, the same Map2 antibody, also labeled only a confined number of dendrites on retinal cryosections (Supplementary Figure S5B). Any attempts to label axons using Tau antibodies failed, as did other immunostainings performed to identify initial axon segments (Ankyrin-G) or pre-and post-synapses (Znp-1, Psd-95, respectively). Staining for the synaptic marker Sv2 did however show extensive labeling throughout the entire retinal cell culture, suggestive for the presence of synapses, and therefore dendrites, within our adult zebrafish retinal culture (data not shown). Although we cannot truly confirm the dendritic specification of neurites in our culture, we are confident that we identified axons in the AC, due to the combination of 450 μm microgrooves and the applied Laminin concentration gradient, (as discussed below) (Dertinger et al., 2002; Taylor et al., 2005, 2010; Cartoni et al., 2017; Virlogeux et al., 2018; Lee et al., 2023). Furthermore, mixed gap43/WT retinal cultures reveal completely isolated RGCs with a defined RGC-like architecture of one long axon-like neurite (the presumable axon) and multiple shorter dendrite-like processes (presumable dendrites) (Supplementary Figure S5C).

### 4.5. Investigation of mitochondrial dynamics in regenerating zebrafish RGCs

#### 4.5.1. Visualization of mitochondria in adult zebrafish RGCs

**Figure 9:**
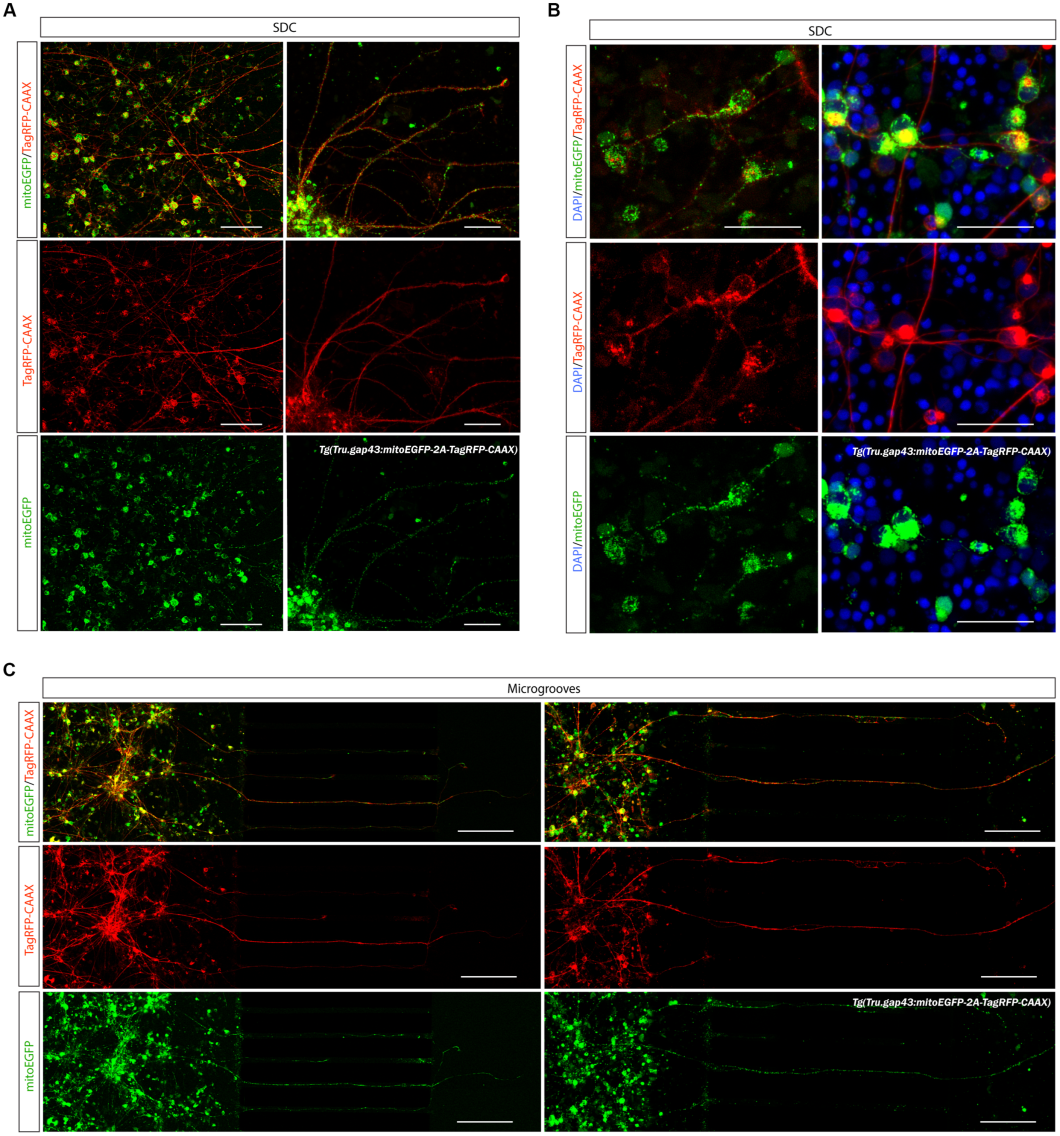
Visualization of mitochondria in adult zebrafish RGCs in microfluidic cultures. The recently developed *Tg(Tru.gap43:mitoEGFP-2A-tagRFP-CAAX)^ulb17^* (gapmito) reporter line enables to study mitochondrial motility in outgrowing and regenerating RGCs. **(A)** Confocal live cell images of gapmito retinal neurons at DIV 3 in an open compartment (SOC450) MFD indicate that outgrowing RGCs express RFP in membranes (middle panels) and EGFP in mitochondria in axons, dendrites and somata (bottom panels). **(B)** Detailed live cell images (left panels), as well as pictures of end-point DAPI-stained cultures of isolated neurons (right panels) in the SDC illustrate that mitochondria in the different neuronal compartments can be visualized with subcellular resolution. **(C)** Likewise in the microgrooves and AC, individual mitochondria can be identified in outgrowing gapmito RGC axons at DIV 3. These results demonstrate how the usefulness of this novel reporter line to characterize mitochondrial dynamics during axonal outgrowth or regeneration. Scale bars: 100 μm **(A,C)**, 50 μm **(B)**. AC, axonal compartment; DIV, *days in vitro*; MFD, microfluidic device; RGC, retinal ganglion cell; SDC, somatodendrtic compartment.

To visualize mitochondria in cultured adult zebrafish RGCs, we used the recently developed *Tg(Tru.gap43:mitoEGFP-2A-tagRFP-CAAX*)*^ulb17^* (gapmito) reporter line, in which outgrowing neurons express both a red fluorescent protein targeted to the plasma membrane, and a green fluorescent protein targeted to the mitochondria (Figure 9A). With this line, individual mitochondria in the different neuronal compartments of RGCs can be identified with subcellular resolution (Figure 9B).

Alternatively, mitochondria in RGCs can be labeled in gap43 adult zebrafish retinal cultures using MitoTracker Red dye supplementation in both the SDC and AC (Supplementary Figure S6A). A disadvantage is that all mitochondria of all cells within the MFD are labeled, which might complicate interpretation of mitochondrial dynamics in outgrowing axons in the AC at DIV 2, as mitochondria inside the conditioned retinal cells seeded in the AC, are also labeled.

#### 4.5.2. Anticipated result: characterization of mitochondrial motility during axonal outgrowth and regeneration

**Figure 10:**
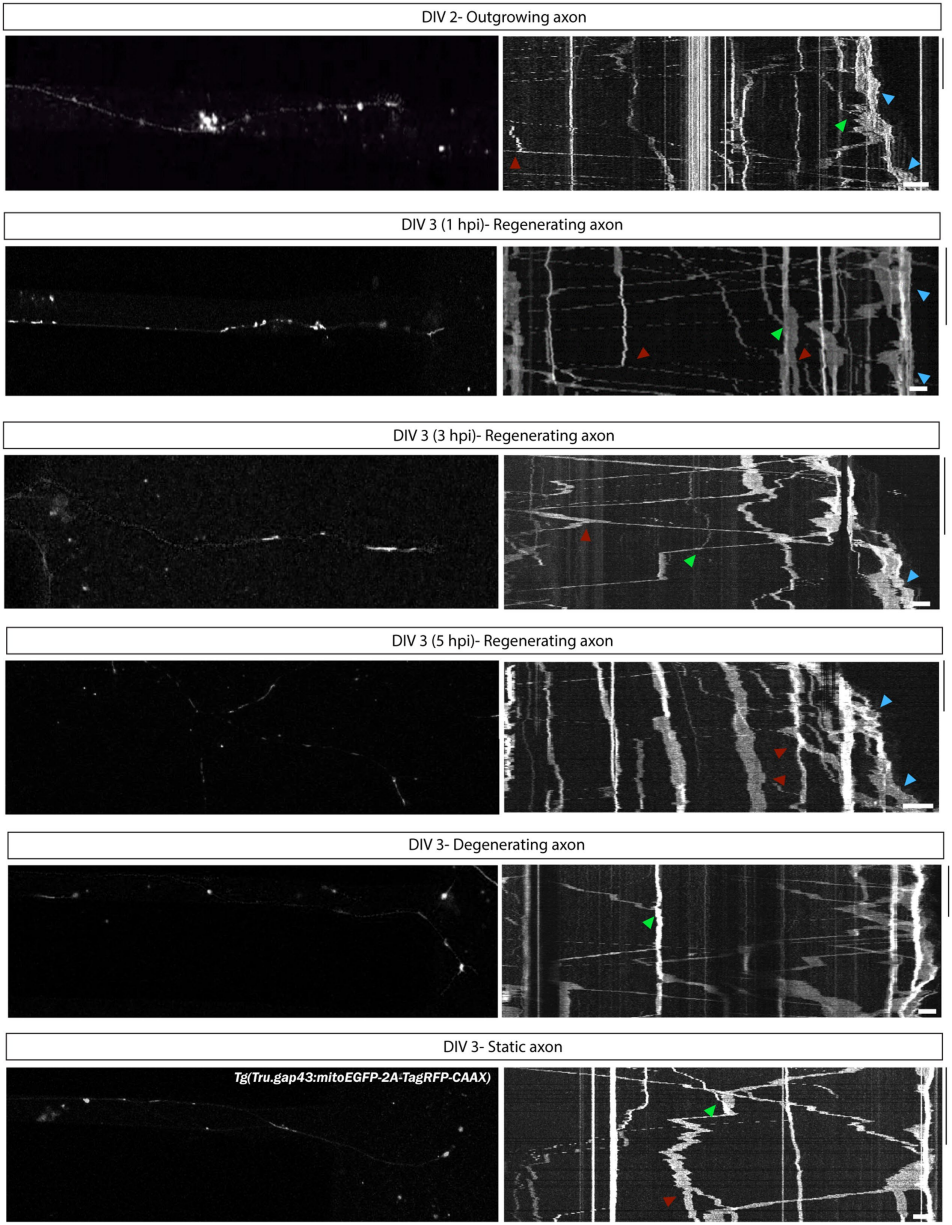
Characterization of mitochondrial motility during axonal outgrowth and regeneration of adult zebrafish RGCs. To characterize mitochondrial dynamics in outgrowing and regenerating RGCs, kymographs of time-lapse live movies, acquired in adult zebrafish retinal neurons cultured in an open compartment (SOC450) MFD, have been generated. Representative confocal still images from time-lapse live recordings in *Tg(Tru.gap43:mitoEGFP-2A-tagRFP-CAAX)^ulb17^* (gapmito) RGC axons and corresponding kymographs illustrate mitochondrial mobility during axonal outgrowth (DIV 2), and in regenerating, static (non-regenerating) and degenerating axons at different time points after axotomy (DIV 3 0–5 hpi). On each kymograph, stationary (vertical lines) as well as motile mitochondria (diagonal lines) can be observed, with mitochondria moving both in anterograde and retrograde directions. Moreover, in outgrowing and regenerating axons, an accumulation of mitochondria can be observed at the distal end of the axon, presumably in the growth cones (blue arrowheads). Of note, these kymographs can also give some first indications on the occurence of additional mitochondrial dynamics like fission (red arrowheads) or fusion (green arrowheads). Kymographs are generated using ImageJ for 20–30 min with an 5-s interval. Scale bars: 10 min (vertical) and 10 μm (horizontal). AC, axonal compartment; DIV, *days in vitro*; hpi, *hours post injury*; MFD, microfluidic device; RGC, retinal ganglion cell.

Mitochondrial mobility during RGC axonal outgrowth and regeneration can be characterized by performing time-lapse live imaging in gapmito retinal cell cultures seeded in microfluidic setups (Figure 9C). Indeed, confocal time-lapse movies acquired in individual RGC axons during outgrowth (DIV 2) and at different time points after injury (DIV 3, 0–5 hpi) in SOC450 MFDs, demonstrate that movement of individual mitochondria can be observed and registered with this setup (Supplementary Movie 7). To describe the movement of individual mitochondria, kymographs are generated from the resulting time-lapse movies (Figure 10; Supplementary Figure S6B). Each kymograph depicts a linear representation of the position of single mitochondria (in the imaged section of the axon) over time. On these kymographs, individual mitochondria appear as lines of which the slope illustrates the direction and velocity of the mitochondria. Representative kymographs of outgrowing, regenerating (0–1, 1–3, 3–5 hpi), static and degenerating axons illustrate how this model can be used to characterize the overall mitochondrial motility during axonal outgrowth and regeneration (Figure 10). Indeed, stationary as well as motile mitochondria can be identified in all conditions, and movement can be observed in both antero-and retrograde directions. First qualitative interpretations illustrate that an accumulation of mitochondria can be detected in axonal growth cones (Figure 10, blue arrowheads) and mitochondrial mobility is higher in outgrowing and regenerating axons as compared to static and degenerating ones.

Several parameters including direction, velocity, location, and percentage of moving mitochondria can be determined by analyzing the resulting kymographs. Quantifying and comparing these parameters in a larger number of time-lapse experiments in all different axonal phenotypes as well as at more time points after injury, will provide insights into the contribution of mitochondrial motility to the various phases of axonal regeneration. For example, comparison of outgrowing (DIV 2) and regenerating neurons (DIV 3), or degenerating and regenerating neurons, may reveal regeneration-specific mitochondrial behaviors.

#### 4.5.3. Anticipated result: characterization of mitochondrial dynamics during axonal regeneration

In addition to mitochondrial motility, these time-lapse movies and resulting kymographs of our gapmito reporter line can also provide suggestive indications of other mitochondrial dynamics during the regenerative process, such as fission and fusion (Figure 10, red and green arrowheads, respectively). However, in future experiments, reporter fish that express a photoconvertible protein in neuronal mitochondria [e.g., *Tg (5kbneurod,mito-mEos)*], might be employed (Mandal et al., 2021). Specific photoconversion of a subset of mitochondria within a small section of the axons will allow to truly characterize transport as well as fusion events in outgrowing and regenerating axons. However, for now, we do not have access to this or any equivalent zebrafish line.

#### 4.5.4. Anticipated result: visualization and quantification of mitochondrial dynamics during dendritic remodeling

Lastly, by using a mixed retinal culture (as described in Section 4.4.1) of gapmito and WT retinal neurons, preliminary data show that mitochondria can be visualized in dendrite-like neurites of individual RGCs in the SDC compartment (Supplementary Figure S7). In future studies, performing time-lapse live imaging and generating and comparing the resulting kymographs in both dendrites and axons of neurons in all aforementioned states will allow to determine how mitochondrial dynamics change in the neuronal compartments during the different phases of axonal and dendritic regrowth.

## 5. Discussion

We previously identified an antagonistic axon-dendrite interplay during spontaneous regeneration of adult zebrafish RGCs and revealed that the distribution and morphology of mitochondria spatiotemporally change in the different neuronal compartments upon ONC (Beckers et al., 2019, 2023c). Based on these observations, we hypothesize that successful injury-induced regeneration requires the temporally organized, local reallocation of energy resources to individual neuronal compartments. In this study, we present a novel adult zebrafish microfluidic *in vitro* model that enables confirming these interactions over time and at single-RGC level.

Firstly, we developed a protocol to successfully culture adult zebrafish retinal neurons. While various publications describe *in vitro* models of primary neurons isolated from embryonic (mammalian and zebrafish) central nervous tissue (Garcia et al., 2002; Zolessi et al., 2006; Chen et al., 2013; Cartoni et al., 2017; Nagendran et al., 2018; Patel et al., 2019; Lenoir et al., 2021), studies using adult neurons are scarce and mostly address short-term cultures with limited cell survival and outgrowth (Romano and Hicks, 2007; Hurst et al., 2020; van Niekerk et al., 2022). Adult mammalian retinal neurons are especially difficult to maintain, due to the limited viability of RGCs upon isolation and the high variety of cell types to sustain (Romano and Hicks, 2007). Of note, the use of adult zebrafish primary neurons *in vitro* is even more scanty (Tapanes-Castillo et al., 2014; Meade et al., 2019), with only one paper reporting on adult zebrafish RGC cultures (Diekmann et al., 2015). Our first attempts, starting from the latter protocol, and using the same gap43 adult zebrafish, yielded limited success as few RGCs survived, and little to no outgrowth was observed. However, by implementing in-house expertise (Van Hove et al., 2015; Van Houcke et al., 2017) and published methodologies for retinal explant and primary cell cultures (Schwartz and Agranoff, 1981; Grozdanov et al., 2010; Veldman et al., 2010), we established a new protocol that enables long-term survival, long-range neurite outgrowth and widespread network formation of adult zebrafish RGCs.

Many of the hurdles faced in our initial trials most likely stem from the different characteristics and requirements of adult zebrafish neurons. Indeed, the composition of most commercially available coating reagents, culture media and supplements, as well as imaging setups, are optimized to maximize mammalian cell viability. The most striking difference might be the requirement for high seeding densities to sustain survival and outgrowth of cultured adult zebrafish retinal neurons. Notably, this density-dependent outgrowth has also been described for an *in vitro* model of adult zebrafish spinal neurons (Meade et al., 2019). In addition, studies using zebrafish primary neurons only describe short-term cultures with a lot of variability in conditions, as well as survival and outgrowth characteristics, highlighting the lack of standardized methods, as compared to more established mammalian *in vitro* models (Chen et al., 2013; Patel et al., 2019).

Once optimized, our protocol was implemented in a commercially available MFD. While current microfluidic models cannot yet fully replicate the complexity of neuronal networks, they provide a simplified recapitulation of *in vivo* microenvironments, as they enable physical and fluidic separation of dendrites and somata from axons. Moreover, due to this fluidic isolation, they allow to precisely monitor and manipulate individual neuronal compartments, which is crucial within our objective to study intraneuronal reallocation of mitochondria in individual RGCs (Harink et al., 2013; Kim et al., 2014; Dai et al., 2022; Lee et al., 2023). As MFDs require only small amounts of medium or cells, reducing not only the cost, but also the number of animals needed to answer specific research questions, microfluidics is becoming an increasingly popular research tool to complement existing *in vivo* and *in vitro* models (Lee et al., 2023). Here, we used open compartment MFDs (SOC450) that allow the creation of a densely seeded neuronal network in the SDC, which sustains fast, spontaneous axonal outgrowth of adult zebrafish RGC axons into the AC after only 3 days in culture (DIV 3).

In most studies, dendrites in MFDs are identified using antibodies, or retrogradely traced using dyes or viral vector approaches in the AC (Taylor et al., 2005; Lenoir et al., 2021). Unfortunately, in our retinal cultures, we were not able to truthfully label dendrites or axons via immunolabeling for Map2 or Tau, respectively. This might be because outgrowing neurites are not sufficiently differentiated to express these markers, or because available antibodies do not (fully) recognize the epitopes of the zebrafish Tau or Map2 proteins. Stainings for Map2 on retinal sections of adult zebrafish also only show limited RGC dendrite labeling, which may reflect cell (sub)types specific differences in the expression of antigens (Beckers et al., 2019). Also, our attempts to differentiate between RGC axons and dendrites using immunostainings for ankyrin-G, Znp-1, Psd-95 or were not fruitful, as such preventing us from reliable identifying the various RGC neurite types. Of note, also viral vector tools remain to be optimized for zebrafish research (Zou et al., 2014; Satou et al., 2022). Although not described in this study, we tested various dyes (e.g., DiI, CellTracker Red, Dextrans, Calcein blue) as well as vector types, promoters, and titers (e.g., AAV2/1-CAG-GFP, AAV2/2-CAG-GFP, AAV2/7-CMV-enh-hSyn-GFP), to label our adult zebrafish RGCs cultures but no successful labeling of dendrites was achieved by any of the tested techniques.

Nevertheless, numerous papers confirmed the successful spatial isolation of axons from dendrites using MFDs. Firstly, the 450 μm barrier length allows axons from neurons seeded in the SDC to enter the AC, whereas dendrites, which grow significantly slower and shorter than axons, normally do not extend more than 400 μm (Taylor et al., 2005; Taylor and Jeon, 2010; Cartoni et al., 2017; Lee et al., 2023). Moreover, as axons preferably grow toward higher concentrations of Laminin, the use of a Laminin concentration gradient from the AC to the SDC successfully targets axonal outgrowth toward the AC (Dertinger et al., 2002; Virlogeux et al., 2018). In addition, mixed, sparsely labeled cultures reveal the distinct architecture of RGCs, with one long axon-like neurite (white arrowhead Supplementary Figure S5), and multiple shorter dendrite-like neurites. These processes are strikingly similar to the neurites identified as dendrites in embryonic cultured zebrafish RGCs after 24 to 28 h in culture, and can therefore be denoted as presumable dendrites (Zolessi et al., 2006). By combining these features, we are confident that we successfully separate axons and dendrite-like neurites in our microfluidic adult zebrafish retinal model.

Our microfluidic setups thus provide an ideal platform to study injury-induced axonal regeneration. Upon vacuum-assisted axotomy at DIV 3, our data revealed that adult zebrafish RGC axons show robust and spontaneous long-range regeneration at 1 day after injury (24 hpi, DIV 4). Due to this fast regeneration, the entire process can be monitored in real-time using time-lapse live imaging within the first 24 h after axotomy. Already within the first hours (0–10 hpi), axonal regeneration as well as dendritic remodeling and mitochondrial dynamics can be monitored and characterized.

Importantly, based on the observation that newly outgrowing axons emerge in the AC in between axotomy and 24 hpi, we want to advise researchers to always consult live-imaging movies in addition to overview pictures to correctly differentiate between newly outgrowing and regenerating axons. Indeed, although most newly outgrowing axons extend in empty grooves, and can thus be identified by comparing images taken before and after injury, some new axons occasionally arise in the same microgroove as regenerating or degenerating axons (Supplementary Movie 1), thereby complicating their identification on confocal still images.

In our model, RGCs axons are repeatedly injured (ONC, isolation, axotomy). Yet, we proved that this conditioning lesioning has no effect on survival, or outgrowth and regenerative potential of the RGC axons, as they still regenerate even after a second *in vitro* axotomy. Moreover, by comparing conditioned and non-conditioned retinas harvested from the same zebrafish, we showed that although non-conditioned RGCs also grow out neurites, and regenerate after injury, outgrowth and regeneration is delayed and highly reduced in number (± 60% and 70% reduction in the number of axons entering the AC, respectively during outgrowth and regeneration).

Another important asset of time-lapse imaging in microfluidic set ups is that it allows visualization of mitochondrial and dendritic changes during axonal regeneration. The characterization and quantification of mitochondrial dynamics in the axons of neuronal cultures in MFDs by time-lapse live imaging and kymography is a well-described technique in literature (Wang and Schwarz, 2009; Chen et al., 2016; Zhou et al., 2016; Cartoni et al., 2017; Huang and Sheng, 2022). Hereto, mitochondria are often identified using transgenic lines, MitoTracker dyes or electroporation/viral delivery of mitochondrial reporter constructs. To our knowledge, this latter methodology has not yet been developed for *in vitro* zebrafish neurons and did also not work in our hands (see above). Despite being successful in our model, we opted not to employ MitoTracker Red, but established a novel transgenic adult zebrafish (gapmito) reporter line that enables constitutive, stable expression specifically in RGCs, our neurons of interest. Moreover, a genetic approach also bypasses the need to label mitochondria after culturing, thereby minimizing manipulation and potential side effects on cell survival and axonal regeneration. This new zebrafish line might be of interest to many other researchers, since to our knowledge, a similar reporter line, with labeled mitochondria in adult zebrafish outgrowing neurons, has not been documented to date.

A first qualitative evaluation of the resulting kymographs obtained from time-lapse imaging in this reporter line illustrates how this model can be used to characterize and compare the overall mitochondrial motility in axons during outgrowth and regeneration. Indeed, extensive movement in both antero-and retrograde directions could be observed and characterized in (re)growing axons. Although it is widely accepted that anterograde transport of mitochondria is essential for axon outgrowth and regeneration, retrograde transport has been mainly considered necessary for the removal of damaged organelles (Zhou et al., 2016). However, a recent study revealed that in zebrafish, retrogradely transported mitochondria are redistributed rather than degraded and are critical to sustain a healthy intracellular distribution of mitochondria that supports neuronal function (Mandal et al., 2021).

Lastly, seeding a mixture of WT and labeled gap43 retinal neurons in the SDC enabled the visualization of individual RGC dendrite-like neurites in the dense cell cultures in the SDC. With this setup, we thus optimized a microfluidic model that allows the visualization of dendritic remodeling during injury-induced axonal regeneration of individual RGCs. Yet, gathering conclusive evidence for both the axon-dendrite and mitochondrial reallocation hypotheses not only requires characterization but also manipulation of RGC dendritic remodeling and (dendritic) mitochondrial dynamics upon axonal injury. Our model is now ready to be used for such compartment-specific manipulation, e.g., via administration of Ca^2+^ or compounds such as mdivi1 in the SDC, respectively inhibiting mitochondrial transport or fission in dendrites, and evaluation of subsequent effects on RGC axonal regeneration in the AC (Taylor and Jeon, 2010; Yao et al., 2019).

While we here describe the methodology to assess mitochondrial dynamics during adult zebrafish RGC regeneration, other compartmentalized intraneuronal molecular processes or vesicular transport (e.g., lysosome trafficking), can also be studied within our established microfluidic model. Furthermore, insights gained throughout the different optimization steps can also be of great value to researchers working with other (non-mammalian) models or cell types for which *in vitro* protocols are not yet available.

With this study, we have successfully established a novel method that enables to isolate and culture neurons from the adult zebrafish retina in a microfluidic set-up and study intraneuronal dynamics in real-time at single-neuron and compartment-specific level during spontaneous injury-induced axonal regrowth. This new model provides a robust platform to gain new insights into the intracellular processes, molecules and metabolites driving successful axonal regeneration in the adult CNS, which in turn may lead to the discovery of novel therapeutic targets to stimulate regeneration in the mammalian CNS.

## Data availability statement

The original contributions presented in the study are included in the article/Supplementary material, further inquiries can be directed to the corresponding author.

## Ethics statement

The animal study was reviewed and approved by KU Leuven Ethical Committee for Animal Experiments (project numbers P121/2018 and P004/2021) and has been performed in accordance with the 2010/63/EU European Communities Council Directive.

## Author contributions

AD and LiM conceived and designed the study. AD performed the experiments and wrote and drafted the manuscript with input from all co-authors. LiM supervised the study. LuM, SB, and AB contributed to conception and design of the study. GS and BV created and designed the transgenic fish line. All the authors contributed to the manuscript and critically reviewed and approved the submitted version of the manuscript.

## Funding

This research was financially supported by the KU Leuven Research Council (C14/18/053 and C14/22/074), the Research Foundation Flanders (FWO) [project: G082221N, fellowships: 1S94218N (AD), 1S42720N (LuM), 1165020N (SB), and 11ZM616N (AB)].

## Conflict of interest

The authors declare that the research was conducted in the absence of any commercial or financial relationships that could be construed as a potential conflict of interest.

## Publisher’s note

All claims expressed in this article are solely those of the authors and do not necessarily represent those of their affiliated organizations, or those of the publisher, the editors and the reviewers. Any product that may be evaluated in this article, or claim that may be made by its manufacturer, is not guaranteed or endorsed by the publisher.

## Figures and Tables

**Table:** 

DAPI	4′,6-diamidino-2-phenylindole
ATP	Adenosine triphosphate
AC	Axonal compartment
BSA	Bovine Serum Albumin
CNS	Central nervous system
CNB	Complete Neurobasal Medium
CL	Conditioning Lesion
cox8a	cytochrome c oxidase subunit 8A
DIV	*Days in vitro*
DMSO	Dimethylsulfoxide
DPBS	Dulbecco’s Phosphate Buffered Saline
hpi	*Hours post injury*
MFD	Microfluidic device
ONC	Optic nerve crush
PBS	Phosphate Buffered Saline
PLL	Poly-L-Lysine
RF	Radio Frequency
ROI	Region of interest
RGC	Retinal ganglion cell
SDC	Somatodendritic compartment
PFA	Paraformaldehyde
PBST	PBS-Triton X-100
PBS	Phosphate buffered saline
PDMS	Polydimethylsiloxane
gap43	*Tg(Tru.gap43:GFP)^mil1^*
gapmito	*Tg(Tru.gap43:mitoEGFP-2A-TagRFP-CAAX)^ulb17^*
WT	Wild type

## References

[ref1] Agostinone J. Alarcon-Martinez L. Gamlin C. Yu W. Q. Wong R. O. L. Di Polo A. (2018). Insulin signalling promotes dendrite and synapse regeneration and restores circuit function after axonal injury. Brain 141, 1963–1980. doi: 10.1093/BRAIN/AWY142, PMID: 29931057 PMC6022605

[ref2] Atkins M. Hazan J. Fassier C. (2022). In vivo live imaging of axonal transport in developing zebrafish axons. Methods Mol. Biol. 2431, 325–350. doi: 10.1007/978-1-0716-1990-2_17/FIGURES/4, PMID: 35412285

[ref3] Bartelt-Kirbach B. Moron M. Glomb M. Beck C. M. Weller M. P. Golenhofen N. (2016). HspB5/αB-crystallin increases dendritic complexity and protects the dendritic arbor during heat shock in cultured rat hippocampal neurons. Cell. Mol. Life Sci. 73, 3761–3775. doi: 10.1007/S00018-016-2219-9, PMID: 27085702 PMC11108385

[ref4] Basu H. Ding L. Pekkurnaz G. Cronin M. Schwarz T. L. (2020). Kymolyzer, a semi-autonomous kymography tool to analyze intracellular motility. Curr. Protoc. Cell Biol. 87:e107. doi: 10.1002/CPCB.107, PMID: 32530579 PMC8989283

[ref01] Becker C. G. Becker T. (2002). Repellent guidance of regenerating optic axons by chondroitin sulfate glycosaminoglycans in zebrafish. J. Neurosci 22, 842–853. 11826114 10.1523/JNEUROSCI.22-03-00842.2002 PMC6758477

[ref5] Becker T. Becker C. G. (2014). Axonal regeneration in zebrafish. Curr. Opin. Neurobiol. 27, 186–191. doi: 10.1016/j.conb.2014.03.019 24769541

[ref6] Beckers A. Bergmans S. Van Dyck A. Moons L. (2023a). Analysis of axonal regrowth and dendritic remodeling after optic nerve crush in adult zebrafish. Methods Mol. Biol. 2636, 163–190. doi: 10.1007/978-1-0716-3012-9_9 36881300

[ref7] Beckers A. Bergmans S. Van Dyck A. Moons L. (2023b). Analysis of visual recovery after optic nerve crush in adult zebrafish. Methods Mol. Biol. 2636, 437–447. doi: 10.1007/978-1-0716-3012-9_24 36881315

[ref8] Beckers A. Masin L. Van Dyck A. Bergmans S. Vanhunsel S. Zhang A. . (2023c). Optic nerve injury-induced regeneration in the adult zebrafish is accompanied by spatiotemporal changes in mitochondrial dynamics. Neural Regen. Res. 18, 219–225. doi: 10.4103/1673-5374.344837, PMID: 35799546 PMC9241429

[ref9] Beckers A. Van Dyck A. Bollaerts I. van Houcke J. Lefevere E. Andries L. . (2019). An antagonistic axon-dendrite interplay enables efficient neuronal repair in the adult zebrafish central nervous system. Mol. Neurobiol. 56, 3175–3192. doi: 10.1007/s12035-018-1292-5, PMID: 30105671

[ref10] Berry M. Ahmed Z. Logan A. (2019). Return of function after CNS axon regeneration: lessons from injury-responsive intrinsically photosensitive and alpha retinal ganglion cells. Prog. Retin. Eye Res. 71, 57–67. doi: 10.1016/J.PRETEYERES.2018.11.006, PMID: 30458239

[ref11] Bohnert S. Schiavo G. (2005). Tetanus toxin is transported in a novel neuronal compartment characterized by a specialized pH regulation. J. Biol. Chem. 280, 42336–42344. doi: 10.1074/jbc.M506750200, PMID: 16236708

[ref12] Cartoni R. Pekkurnaz G. Wang C. Schwarz T. L. He Z. (2017). A high mitochondrial transport rate characterizes CNS neurons with high axonal regeneration capacity. PLoS One 12:e0184672. doi: 10.1371/JOURNAL.PONE.0184672, PMID: 28926622 PMC5604968

[ref13] Cavallucci V. Bisicchia E. Cencioni M. T. Ferri A. Latini L. Nobili A. . (2014). Acute focal brain damage alters mitochondrial dynamics and autophagy in axotomized neurons. Cell Death Dis. 5:e1545. doi: 10.1038/cddis.2014.511, PMID: 25429622 PMC4260762

[ref14] Chen Z. Lee H. Henle S. J. Cheever T. R. Ekker S. C. Henley J. R. (2013). Primary neuron culture for nerve growth and axon guidance studies in zebrafish (*Danio rerio*). PLoS One 8:e57539. doi: 10.1371/journal.pone.0057539, PMID: 23469201 PMC3587632

[ref15] Chen M. Li Y. Yang M. Chen X. Chen Y. Yang F. . (2016). A new method for quantifying mitochondrial axonal transport. Protein Cell 7, 804–819. doi: 10.1007/S13238-016-0268-3, PMID: 27225265 PMC5084152

[ref16] Cheng X. T. Huang N. Sheng Z. H. (2022). Programming axonal mitochondrial maintenance and bioenergetics in neurodegeneration and regeneration. Neuron 110, 1899–1923. doi: 10.1016/J.NEURON.2022.03.015, PMID: 35429433 PMC9233091

[ref17] Claes M. Santos J. R. F. Masin L. Cools L. Davis B. M. Arckens L. . (2021). A fair assessment of evaluation tools for the murine microbead occlusion model of glaucoma. Int. J. Mol. Sci. 22:5633. doi: 10.3390/IJMS22115633/S1, PMID: 34073191 PMC8199180

[ref18] Dai C. Liu X. Tang R. He J. Arai T. (2022). A review on microfluidic platforms applied to nerve regeneration. Appl. Sci. 12:3534. doi: 10.3390/APP12073534

[ref19] Della Santina L. Inman D. M. Lupien C. B. Horner P. J. Wong R. O. (2013). Differential progression of structural and functional alterations in distinct retinal ganglion cell types in a mouse model of glaucoma. J. Neurosci. 33, 17444–17457. doi: 10.1523/JNEUROSCI.5461-12.2013, PMID: 24174678 PMC3812509

[ref20] Dertinger S. K. W. Jiang X. Li Z. Murthy V. N. Whitesides G. M. (2002). Gradients of substrate-bound laminin orient axonal specification of neurons. Proc. Natl. Acad. Sci. U. S. A. 99, 12542–12547. doi: 10.1073/PNAS.192457199, PMID: 12237407 PMC130496

[ref21] Dhara S. P. Rau A. Flister M. J. Recka N. M. Laiosa M. D. Auer P. L. . (2019). Cellular reprogramming for successful CNS axon regeneration is driven by a temporally changing cast of transcription factors. Sci. Rep. 9, 14198–14112. doi: 10.1038/s41598-019-50485-6, PMID: 31578350 PMC6775158

[ref22] Dhara S. P. Udvadia A. J. (2023). Profiling dynamic changes in DNA accessibility during axon regeneration after optic nerve crush in adult zebrafish. Methods Mol. Biol. 2636, 323–341. doi: 10.1007/978-1-0716-3012-9_18 36881309

[ref23] Diekmann H. Kalbhen P. Fischer D. (2015). Characterization of optic nerve regeneration using transgenic zebrafish. Front. Cell. Neurosci. 9:118. doi: 10.3389/fncel.2015.00118, PMID: 25914619 PMC4391235

[ref02] Elsaeidi F. Bemben M. A. Zhao X. F. Goldman D. (2014). Jak/Stat signaling stimulates zebrafish optic nerve regeneration and overcomes the inhibitory actions of Socs3 and Sfpq. J Neurosci, 34, 2632–2644. doi: 10.1523/JNEUROSCI.3898-13.2014 24523552 PMC3921430

[ref24] Emily M. F. Agrawal L. Barzaghi P. Otsuki M. Terenzio M. (2022). Use of microfluidics chambers to image axonal transport in adult sensory neurons. Methods Mol. Biol. 2431, 271–288. doi: 10.1007/978-1-0716-1990-2_14/FIGURES/5, PMID: 35412282

[ref25] Fawcett J. W. (2020). The struggle to make CNS axons regenerate: why has it been so difficult? Neurochem. Res. 45, 144–158. doi: 10.1007/S11064-019-02844-Y, PMID: 31388931 PMC6942574

[ref26] Ferreira T. A. Blackman A. V. Oyrer J. Jayabal S. Chung A. J. Watt A. J. . (2014). Neuronal morphometry directly from bitmap images. Nature Methods 11, 982–984. doi: 10.1038/nmeth.3125, PMID: 25264773 PMC5271921

[ref27] Fleisch V. C. Fraser B. Allison W. T. (2011). Investigating regeneration and functional integration of CNS neurons: lessons from zebrafish genetics and other fish species. Biochim. Biophys. Acta 1812, 364–380. doi: 10.1016/j.bbadis.2010.10.012, PMID: 21044883

[ref28] Garcia M. Forster V. Hicks D. Vecino E. (2002). Effects of Muller glia on cell survival and 1282 neuritogenesis in adult porcine retina in vitro. Invest. Ophthalmol. Vis. Sci. 43, 3735–3743. PMID: 12454045

[ref29] Grozdanov V. Muller A. Sengottuvel V. Leibinger M. Fischer D. (2010). A method for preparing primary retinal cell cultures for evaluating the neuroprotective and neuritogenic effect of factors on axotomized mature CNS neurons. Curr. Protoc. Neurosci. Chapter 3:Unit3.22. doi: 10.1002/0471142301.ns0322s53 20938922

[ref30] Han S. M. Baig H. S. Hammarlund M. (2016). Mitochondria localize to injured axons to support regeneration. Neuron 92, 1308–1323. doi: 10.1016/J.NEURON.2016.11.025, PMID: 28009276 PMC5364819

[ref31] Han Q. Xie Y. Ordaz J. D. Huh A. J. Huang N. Wu W. . (2020). Restoring cellular energetics promotes axonal regeneration and functional recovery after spinal cord injury. Cell Metab. 31, 623–641.e8. doi: 10.1016/J.CMET.2020.02.002, PMID: 32130884 PMC7188478

[ref32] Harink B. Le Gac S. Truckenmüller R. Van Blitterswijk C. Habibovic P. (2013). Regeneration-on-a-chip? The perspectives on use of microfluidics in regenerative medicine. Lab Chip 13, 3512–3528. doi: 10.1039/C3LC50293G, PMID: 23877890

[ref33] Huang N. Sheng Z. H. (2022). Microfluidic devices as model platforms of CNS injury-ischemia to study axonal regeneration by regulating mitochondrial transport and bioenergetic metabolism. Cell Regen. 11:33. doi: 10.1186/S13619-022-00138-3, PMID: 36184647 PMC9527262

[ref34] Hurst J. Fietz A. Tsai T. Joachim S. C. Schnichels S. (2020). Organ cultures for retinal diseases. Front. Neurosci. 14:1150. doi: 10.3389/FNINS.2020.583392/BIBTEX PMC7724035 33324149

[ref35] Kim S. Park J. Han A. Li J. (2014). Microfluidic systems for axonal growth and regeneration research. Neural Regen. Res. 9, 1703–1705. doi: 10.4103/1673-5374.143412, PMID: 25422629 PMC4238156

[ref36] Kulkarni V. A. Firestein B. L. (2012). The dendritic tree and brain disorders. Mol. Cell. Neurosci. 50, 10–20. doi: 10.1016/J.MCN.2012.03.005 22465229

[ref37] Kwan K. M. Fujimoto E. Grabher C. Mangum B. D. Hardy M. E. Campbell D. S. . (2007). The Tol2kit: a multisite gateway-based construction kit for Tol2 transposon transgenesis constructs. Dev. Dyn. 236, 3088–3099. doi: 10.1002/DVDY.21343, PMID: 17937395

[ref04] Landreth G. E. Agranoff B. W. (1976). Explant culture of adult goldfish retina: effect of prior optic nerve crush. Brain Research, 118, 299–303. doi: 10.1016/0006-8993(76)90714-9 1000292

[ref38] Lee D. Yang K. Xie J. Lee D. Xie J. Yang K. (2023). Advances in nerve injury models on a Chip. Adv. Biol.:2200227. doi: 10.1002/ADBI.202200227, PMID: 36709421

[ref39] Lenoir S. Genoux A. Agasse F. Saudou F. Humbert S. (2021). Recreating mouse cortico-hippocampal neuronal circuit in microfluidic devices to study BDNF axonal transport upon glucocorticoid treatment. STAR Protocols 2:100382. doi: 10.1016/J.XPRO.2021.100382 33748784 PMC7972978

[ref40] Mandal A. Wong H. T. C. Pinter K. Mosqueda N. Beirl A. Lomash R. M. . (2021). Retrograde mitochondrial transport is essential for organelle distribution and health in zebrafish neurons. J. Neurosci. 41, 1371–1392. doi: 10.1523/JNEUROSCI.1316-20.2020, PMID: 33376159 PMC7896009

[ref41] Mar F. M. Simões A. R. Leite S. Morgado M. M. Santos T. E. Rodrigo I. S. . (2014). CNS axons globally increase axonal transport after peripheral conditioning. J. Neurosci. Off. J. Soc. Neurosci. 34, 5965–5970. doi: 10.1523/JNEUROSCI.4680-13.2014, PMID: 24760855 PMC6608291

[ref42] Meade M. E. Roginsky J. E. Schulz J. R. (2019). Primary cell culture of adult zebrafish spinal neurons for electrophysiological studies. J. Neurosci. Methods 322, 50–57. doi: 10.1016/J.JNEUMETH.2019.04.011, PMID: 31028770 PMC6530804

[ref43] Nagendran T. Poole V. Harris J. Taylor A. M. (2018). Use of pre-assembled plastic microfluidic chips for compartmentalizing primary murine neurons. J. Vis. Exp.:e58421. doi: 10.3791/58421, PMID: 30451222 PMC6420830

[ref44] Neumann S. Chassefeyre R. Campbell G. E. Encalada S. E. (2017). Kymo analyzer: a software tool for the quantitative analysis of intracellular transport in neurons. Traffic 18, 71–88. doi: 10.1111/TRA.12456, PMID: 27770501 PMC5473519

[ref45] Otsuki L. Brand A. H. (2020). Quiescent neural stem cells for brain repair and regeneration: lessons from model systems. Trends Neurosci. 43, 213–226. doi: 10.1016/J.TINS.2020.02.002, PMID: 32209453

[ref46] Padilla S. Lyerly D. (1986). Effects of hypothermia on the in vivo measurement of rapid axonal transport in the rat: a cautionary note. J. Neurochem. 46, 1227–1230. doi: 10.1111/J.1471-4159.1986.TB00642.X, PMID: 2419507

[ref47] Park J. W. Vahidi B. Taylor A. M. Rhee S. W. Jeon N. L. (2006). Microfluidic culture platform for neuroscience research. Nat. Protoc. 1, 2128–2136. doi: 10.1038/nprot.2006.316 17487204

[ref48] Patel B. B. Clark K. L. Kozik E. M. Dash L. Kuhlman J. A. Sakaguchi D. S. (2019). Isolation and culture of primary embryonic zebrafish neural tissue. J. Neurosci. Methods 328:108419. doi: 10.1016/J.JNEUMETH.2019.108419, PMID: 31472190

[ref49] Patrón L. A. Zinsmaier K. E. (2016). Mitochondria on the road to power axonal regeneration. Neuron 92, 1152–1154. doi: 10.1016/J.NEURON.2016.12.007, PMID: 28009268

[ref50] Peterson S. L. Benowitz L. I. (2018). Mammalian dendritic regrowth: a new perspective on neural repair. Brain 141, 1891–1894. doi: 10.1093/BRAIN/AWY165, PMID: 30053179 PMC6658712

[ref51] Rasmussen J. P. Sagasti A. (2016). Learning to swim, again: axon regeneration in fish. Exp. Neurol. 287, 318–330. doi: 10.1016/j.expneurol.2016.02.022, PMID: 26940084

[ref52] Romano C. Hicks D. (2007). Adult retinal neuronal cell culture. Prog. Retin. Eye Res. 26, 379–397. doi: 10.1016/J.PRETEYERES.2007.03.001 17482863

[ref53] Satou C. Neve R. L. Oyibo H. K. Zmarz P. Huang K. H. Bouldoires E. A. . (2022). A viral toolbox for conditional and transneuronal gene expression in zebrafish. elife 11:e77153. doi: 10.7554/ELIFE.77153, PMID: 35866706 PMC9307271

[ref03] Schwartz M. (2004). Optic nerve crush: protection and regeneration. Brain Res. Bull. 62, 467–471. doi: 10.1016/s0361-9230(03)00076-5 15036559

[ref54] Schwartz M. Agranoff B. W. (1981). Outgrowth and maintenance of neurites from cultured goldfish retinal ganglion cells. Brain Res. 206, 331–343. doi: 10.1016/0006-8993(81)90535-7, PMID: 7011471

[ref55] Shekari A. Fahnestock M. (2022). Retrograde axonal transport of neurotrophins in basal forebrain cholinergic neurons. Methods Mol. Biol. 2431, 249–270. doi: 10.1007/978-1-0716-1990-2_13/FIGURES/6, PMID: 35412281

[ref56] Tapanes-Castillo A. Shabazz F. S. Mboge M. Y. Vajn K. Oudega M. Plunkett J. A. (2014). Characterization of a novel primary culture system of adult zebrafish brainstem cells. J. Neurosci. Methods 223, 11–19. doi: 10.1016/J.JNEUMETH.2013.11.022, PMID: 24316294

[ref57] Taylor A. M. Blurton-Jones M. Rhee S. W. Cribbs D. H. Cotman C. W. Jeon N. L. (2005). A microfluidic culture platform for CNS axonal injury, regeneration and transport. Nat. Methods 2, 599–605. doi: 10.1038/nmeth777, PMID: 16094385 PMC1558906

[ref58] Taylor A. M. Dieterich D. C. Ito H. T. Kim S. A. Schuman E. M. (2010). Microfluidic local perfusion chambers for the visualization and manipulation of synapses. Neuron 66, 57–68. doi: 10.1016/J.NEURON.2010.03.022, PMID: 20399729 PMC2879052

[ref59] Taylor A. M. Jeon N. L. (2010). Micro-scale and microfluidic devices for neurobiology. Curr. Opin. Neurobiol. 20, 640–647. doi: 10.1016/J.CONB.2010.07.011, PMID: 20739175

[ref60] Udvadia A. J. (2008). 3.6 kb genomic sequence from Takifugu capable of promoting axon growth-associated gene expression in developing and regenerating zebrafish neurons. Gene Expr. Patterns 8, 382–388. doi: 10.1016/j.gep.2008.05.002, PMID: 18599366 PMC2603057

[ref61] Van Dyck A. Bollaerts I. Beckers A. Vanhunsel S. Glorian N. Houcke J. . (2021). Müller glia–myeloid cell crosstalk accelerates optic nerve regeneration in the adult zebrafish. Glia 69, 1444–1463. doi: 10.1002/glia.23972, PMID: 33502042

[ref62] Van Houcke J. Bollaerts I. Geeraerts E. Davis B. Beckers A. Van Hove I. . (2017). Successful optic nerve regeneration in the senescent zebrafish despite age-related decline of cell intrinsic and extrinsic response processes. Neurobiol. Aging 60, 1–10. doi: 10.1016/J.NEUROBIOLAGING.2017.08.013, PMID: 28917662

[ref63] Van Hove I. Lefevere E. Moons L. (2015). ROCK inhibition as a novel potential strategy for axonal regeneration in optic neuropathies. Neural Regen. Res. 10, 1949–1950. doi: 10.4103/1673-5374.172311, PMID: 26889182 PMC4730818

[ref64] van Niekerk E. A. Kawaguchi R. Marques de Freria C. Groeniger K. Marchetto M. C. Dupraz S. . (2022). Methods for culturing adult CNS neurons reveal a CNS conditioning effect. Cell Rep. Methods 2:100255. doi: 10.1016/J.CRMETH.2022.100255, PMID: 35880023 PMC9308166

[ref65] Varadarajan S. G. Hunyara J. L. Hamilton N. R. Kolodkin A. L. Huberman A. D. (2022). Central nervous system regeneration. Cells 185, 77–94. doi: 10.1016/J.CELL.2021.10.029 PMC10896592 34995518

[ref66] Veldman M. B. Bemben M. A. Goldman D. (2010). Tuba1a gene expression is regulated by KLF6/7 and is necessary for CNS development and regeneration in zebrafish. Mol. Cell. Neurosci. 43, 370–383. doi: 10.1016/j.mcn.2010.01.004, PMID: 20123021 PMC2837137

[ref67] Verreet T. Weaver C. J. Hino H. Hibi M. Poulain F. E. (2019). Syntaphilin-mediated docking of mitochondria at the growth cone is dispensable for axon elongation in vivo. ENeuro 6. doi: 10.1523/ENEURO.0026-19.2019, PMID: 31481398 PMC6751374

[ref68] Virlogeux A. Moutaux E. Christaller W. Genoux A. Bruyère J. Fino E. . (2018). Reconstituting corticostriatal network on-a-chip reveals the contribution of the presynaptic compartment to Huntington’s disease. Cell Rep. 22, 110–122. doi: 10.1016/J.CELREP.2017.12.013, PMID: 29298414

[ref69] Wang X. Schwarz T. L. (2009). Imaging axonal transport of mitochondria. Methods Enzymol. 457:319. doi: 10.1016/S0076-6879(09)05018-6, PMID: 19426876 PMC2996865

[ref70] Williams P. R. Benowitz L. I. Goldberg J. L. He Z. (2020). Axon regeneration in the mammalian optic nerve. Annu. Rev. Vis. Sci. 6, 195–213. doi: 10.1146/annurev-vision-022720-094953 32936739

[ref71] Xu Y. Chen M. Hu B. Huang R. Hu B. (2017). In vivo imaging of mitochondrial transport in single-axon regeneration of zebrafish mauthner cells. Front. Cell. Neurosci. 11:4. doi: 10.3389/FNCEL.2017.00004/BIBTEX 28174522 PMC5258718

[ref72] Yao N. Wang C. Hu N. Li Y. Liu M. Lei Y. . (2019). Inhibition of PINK1/Parkin-dependent mitophagy sensitizes multidrug-resistant cancer cells to B5G1, a new betulinic acid analog. Cell Death Dis. 10, 232–216. doi: 10.1038/s41419-019-1470-z, PMID: 30850585 PMC6408511

[ref73] Zhou B. Yu P. Lin M. Y. Sun T. Chen Y. Sheng Z. H. (2016). Facilitation of axon regeneration by enhancing mitochondrial transport and rescuing energy deficits. J. Cell Biol. 214, 103–119. doi: 10.1083/JCB.201605101/VIDEO-6, PMID: 27268498 PMC4932375

[ref74] Zolessi F. R. Poggi L. Wilkinson C. J. Chien C. B. Harris W. A. (2006). Polarization and orientation of retinal ganglion cells in vivo. Neural Dev. 1:2. doi: 10.1186/1749-8104-1-2, PMID: 17147778 PMC1636330

[ref75] Zou M. De Koninck P. Neve R. L. Friedrich R. W. (2014). Fast gene transfer into the adult zebrafish brain by herpes simplex virus 1 (HSV-1) and electroporation: methods and optogenetic applications. Front. Neural Circuits 8:41. doi: 10.3389/FNCIR.2014.00041/BIBTEX 24834028 PMC4018551

