# Supplementary material for: A new microfluidic model to study dendritic remodeling and mitochondrial dynamics during axonal regeneration of adult zebrafish retinal neurons
Source: Front Mol Neurosci. 2023 Jun 15;16:1196504. doi: 10.3389/fnmol.2023.1196504 (PMC10307971; doi:10.3389/fnmol.2023.1196504)
Supplement: Supplementary file 8 [file Data_Sheet_1.pdf]

Supplementary Material

A new microfluidic model to study dendritic remodeling and mitochondrial dynamics during axonal regeneration of adult zebrafish retinal neurons

Annelies Van Dyck, Luca Masin, Steven Bergmans, Giel Schevenels, An Beckers, Benoit Vanhollebeke, Lieve Moons*

*** Correspondence:** Lieve Moons - lieve.moons@kuleuven.be

1 Supplementary Figures and Movies

1.1 Supplementary Figures

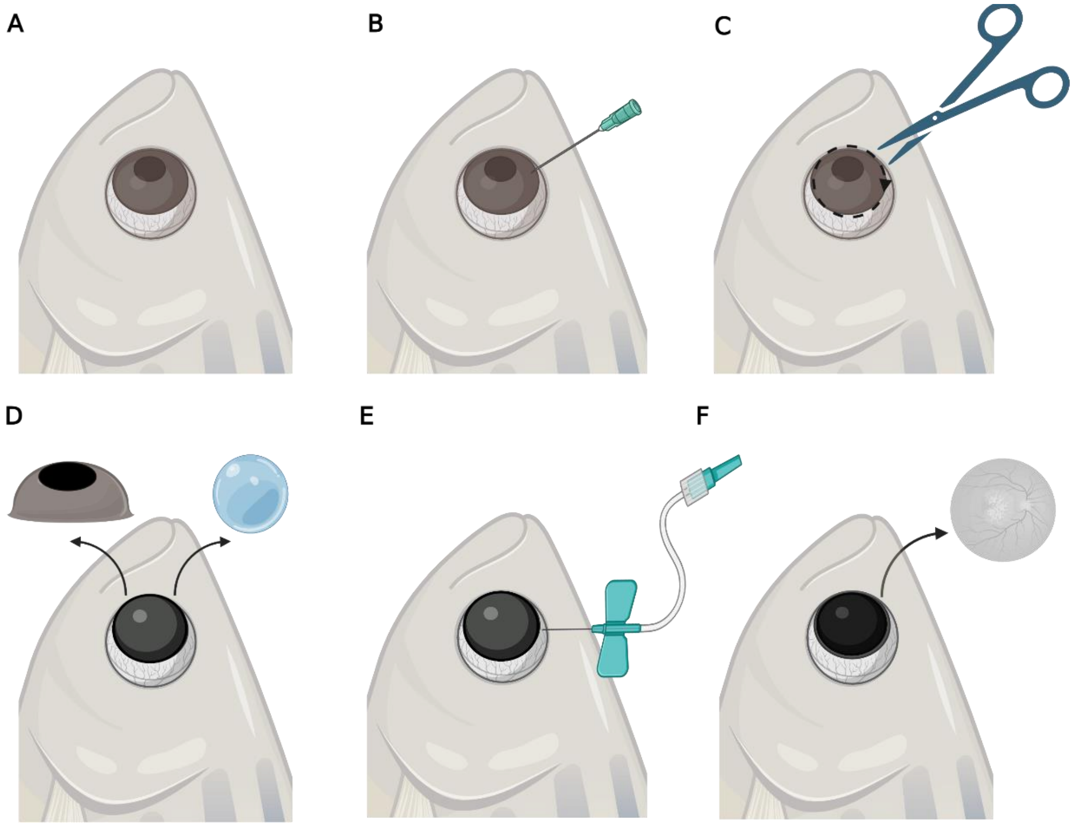

Supplementary Figure 1: Schematic illustration of the retina isolation procedure. **A-F** To dissect the retina at DIV 0, the fish is transferred into the euthanasia solution (0.1% tricaine) and placed on a sterile surgical gauze (A). A small hole is made at the dorsal side of the crushed eye using a sharp needle (B). Via this hole, the sclera and cornea are cut away (C), whereafter these tissues and the lens can be removed to expose the retina (D). Next, the retina is cleared from the surrounding tissue using a winged needle connected to a syringe filled with fish medium (E). Lastly, the optic nerve is cut and the retina can be collected using forceps (F). DIV: days *in vitro*, ONC: optic nerve crush.

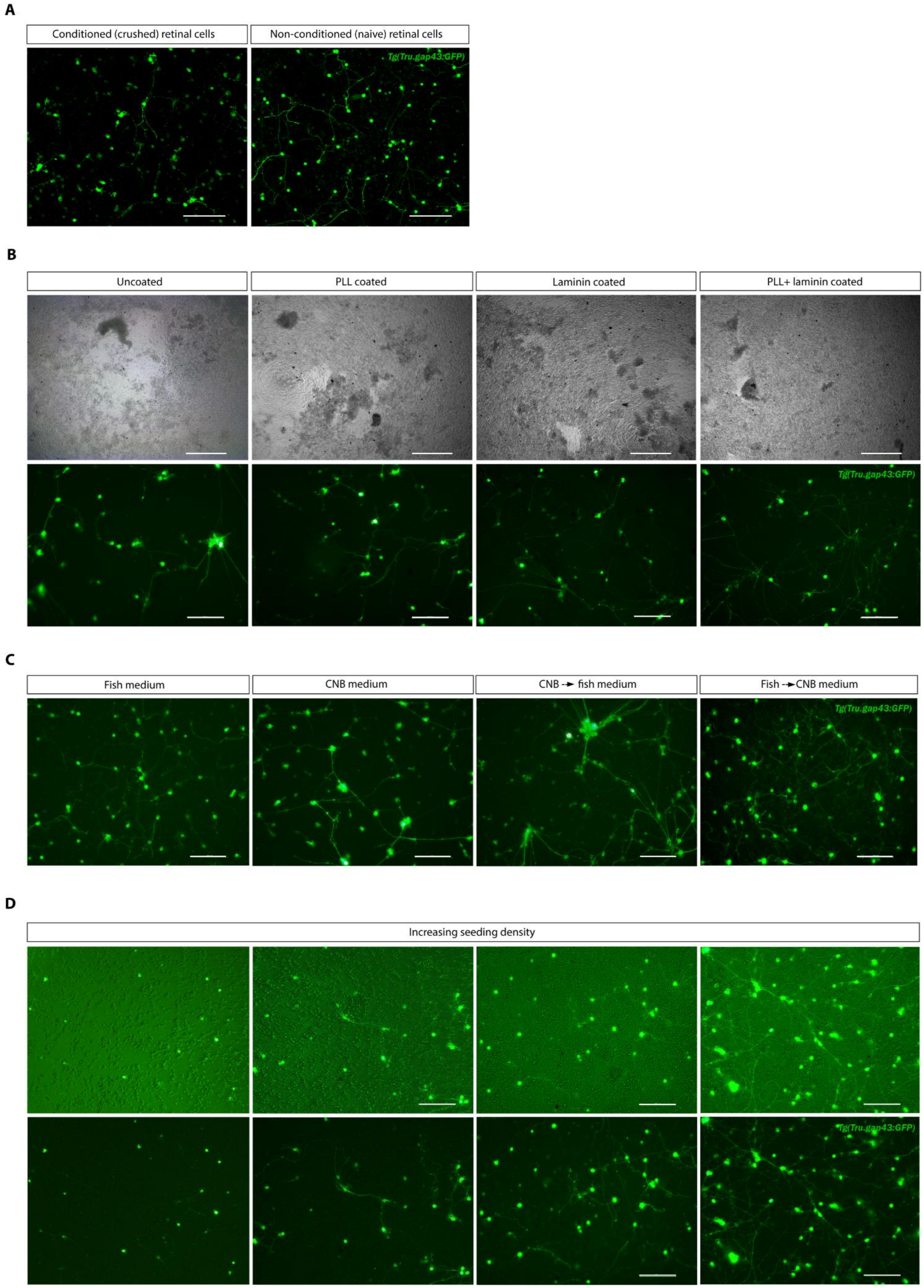
A
Conditioned (crushed) retinal cells
Non-conditioned (naive) retinal cells
Tg(Tru.gap43:GFP)
B
Uncoated
PLL coated
Laminin coated
PLL+ laminin coated
Tg(Tru.gap43:GFP)
C
Fish medium
CNB medium
CNB -> fish medium
Fish -> CNB medium
Tg(Tru.gap43:GFP)
D
Increasing seeding density
Tg(Tru.gap43:GFP)

Supplementary Figure 2: Establishment of an adult zebrafish retinal cell culture protocol. Various optimization steps resulted in our newly established, standardized protocol that enables long-term culture of adult zebrafish retinal neurons. (**A)** Performing an ONC prior to cell isolation (DIV -2) primes the adult zebrafish RGCs for regeneration *in vitro*. This conditioning step results in more and faster outgrowth of adult *Tg(Tru.gap43:GFP)*^{mil1} (gap43) zebrafish RGCs at DIV 3, compared to non-conditioned (uncrushed) retinal neurons. (**B)** The use of sequential overnight PLL and Laminin coating of the glass cover slips reduces cell clumping as a result of detaching cells (brightfield pictures, top panels) and greatly improves outgrowth (fluorescence pictures, bottom panels) compared to uncoated or single PLL or Laminin coated surfaces at DIV 3. (**C)** Seeding cells in fresh fish medium at DIV 0, and transitioning to CNB medium from DIV 1 onwards results in improved outgrowth at DIV 3, compared to the shorter, or more rugged outgrowth obtained when neurons are cultured in solely fish or CNB medium, or when the order is reverted. (**D)** Increasing the seeding density (brightfield/fluorescence overlay pictures in top panels) to a final density of 22000 cells/mm² not only results in more network formation at DIV 3, but also increases survival and outgrowth of individual neurons (fluorescence pictures, bottom panels). By combining all these optimized parameters, a successful protocol was established to sustain a long-term outgrowing adult zebrafish retinal culture. Scale bars: 100 µm (A, B (bottom panels), C, D), 50 µm (B (top panels)). DIV: days *in vitro*, ONC: optic nerve crush, PLL: Poly-L-lysine, RGC: retinal ganglion cell.

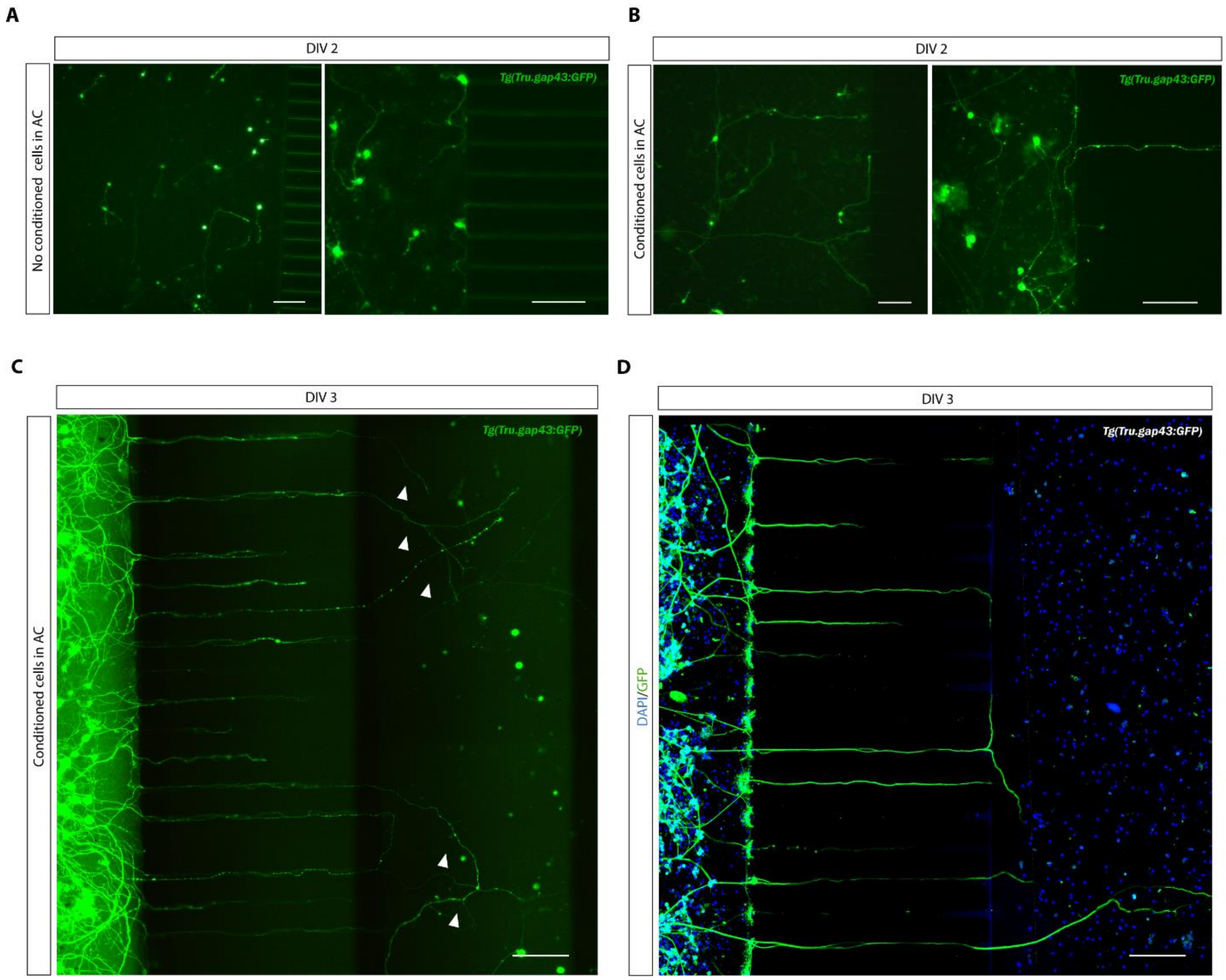

Supplementary figure 3: Improved outgrowth of adult zebrafish RGCs upon addition of conditioned cells in the AC. Addition of conditioned (crushed) retinal cells in the AC results in improved outgrowth of adult zebrafish RGCs axons into the microgrooves in both SND450 and SOC450 MFDs. (**A)** Live cell images taken at DIV 2 disclose that when conditioned cells are added in the AC wells and channel of a SND450 MFD, axonal sprouting of adult *Tg(Tru.gap43:GFP)^{mil1}* (gap43) zebrafish RGCs seeded in the SDC is oriented towards the grooves with some axons growing into the microgrooves towards the AC. (**B)** In contrast, in the conditions without cells in the AC, axonal sprouting within the SDC is more randomly oriented and (almost) no axons grow into the grooves at DIV 2. (**C)** In open compartment SOC450 MFDs, conditioned cells are always seeded into the AC. Confocal live images at DIV 3 illustrate that this addition promotes outgrowth of gap43 RGC axons, many long axons grow into the AC and towards the conditioned cells in the AC channel (white arrowheads).(**D)** Confocal images of DAPI-stained fixed gap43 retinal cultures illustrate the presence of WT conditioned retinal cells in the AC of an open compartment (SOC450) MFD at DIV 3. Scale bars: 100 µm. AC: axonal compartment, DIV: *days in vitro*, RGC: retinal ganglion cell, SDC: somatodendritic compartment.

A

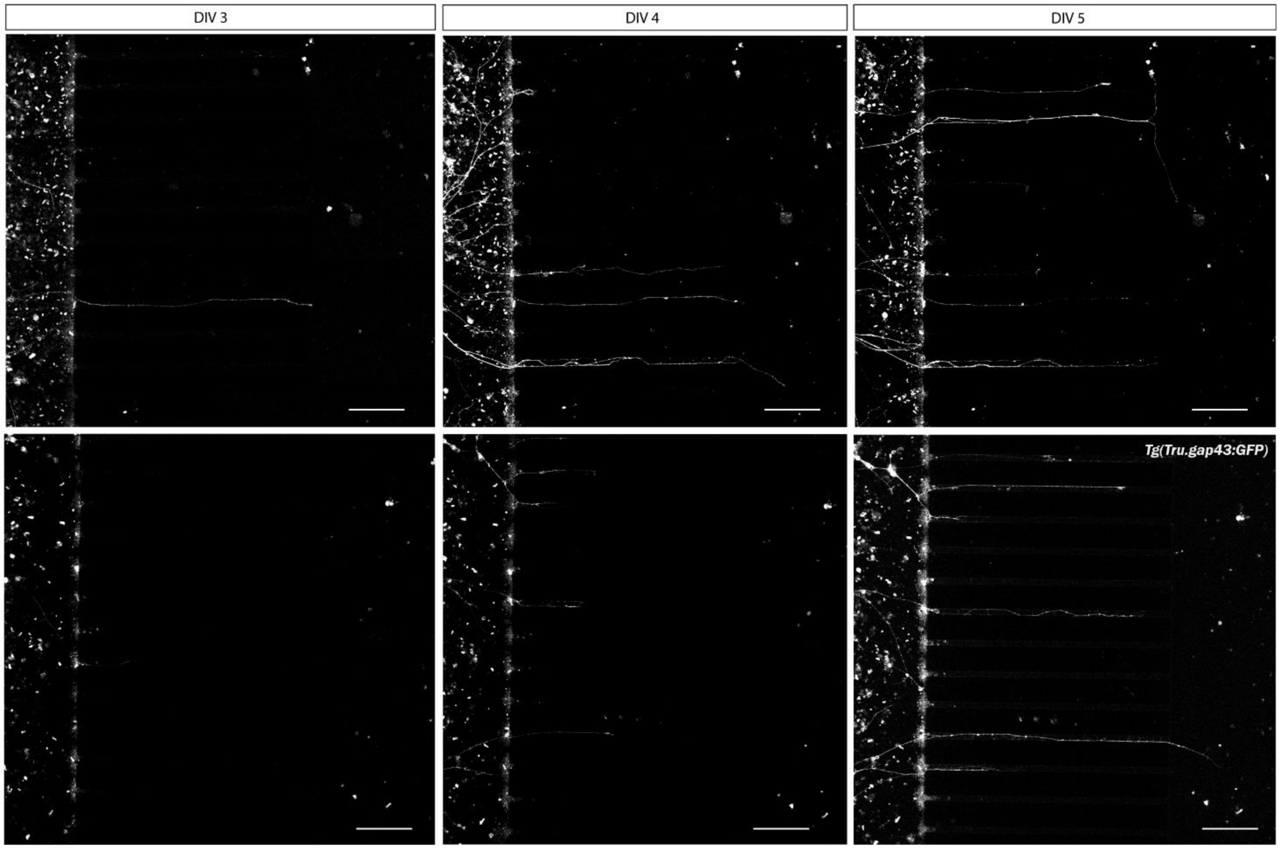
DIV 3
DIV 4
DIV 5
Tg(Tru.gap43:GFP)

B

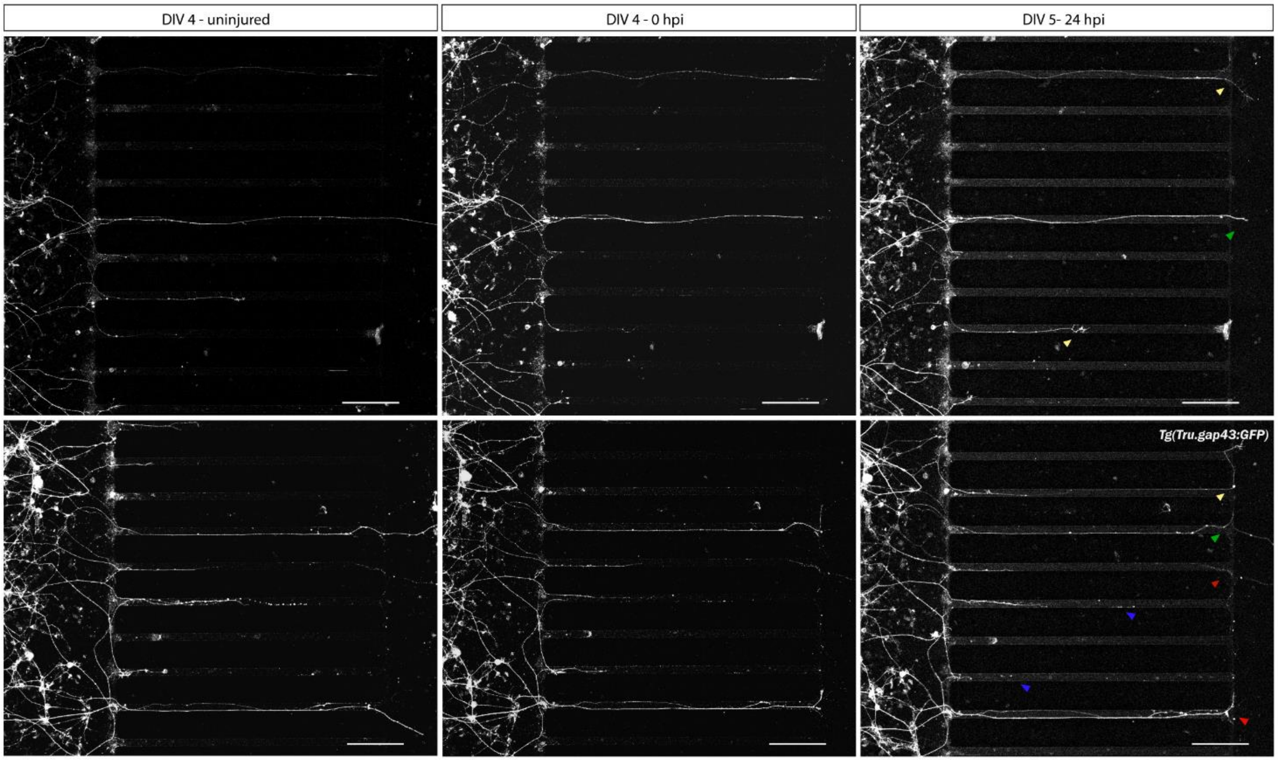
DIV 4 - uninjured
DIV 4 - 0 hpi
DIV 5- 24 hpi
Tg(Tru.gap43:GFP)

Supplementary Figure 4: Axonal outgrowth and regeneration of non-conditioned retinal neurons in a microfluidic setup. Without an *in vivo* conditioning lesion (ONC), cultured adult zebrafish retinal neurons grow out and regenerate after *in vitro* axotomy, but at a slower pace, and with reduced numbers compared to retinal neurons conditioned two days before seeding (figure 5). (**A)** Representative confocal live cell images of adult *Tg(Tru.gap43:GFP)*^{mil1} (gap43) zebrafish retinal neurons in an open compartment (SOC450) taken at DIV 3-5 disclose that RGC axons start to grow out into the microgrooves from DIV 3 onwards, and some axons reach the AC at DIV 4-5. In conditioned cultures, numerous RGC axons reach the AC at DIV 3 (see figure 5). (**B)** Upon axotomy, performed at DIV 4, regenerating (green arrowheads), as well as newly outgrowing (yellow arrowheads), degenerating (blue arrowheads) and static (red arrowheads) axons can be observed in the microgrooves and AC at 24 hpi (DIV 5), but the numbers and length of these axons is more limited, as compared to what is observed in conditioned cultures (see figure 5). Scale bar: 100 µm. AC: axonal compartment, DIV: days *in vitro*; hpi, *hours post injury*, MFD: microfluidic device, ONC: optic nerve crush, RGC: retinal ganglion cell.

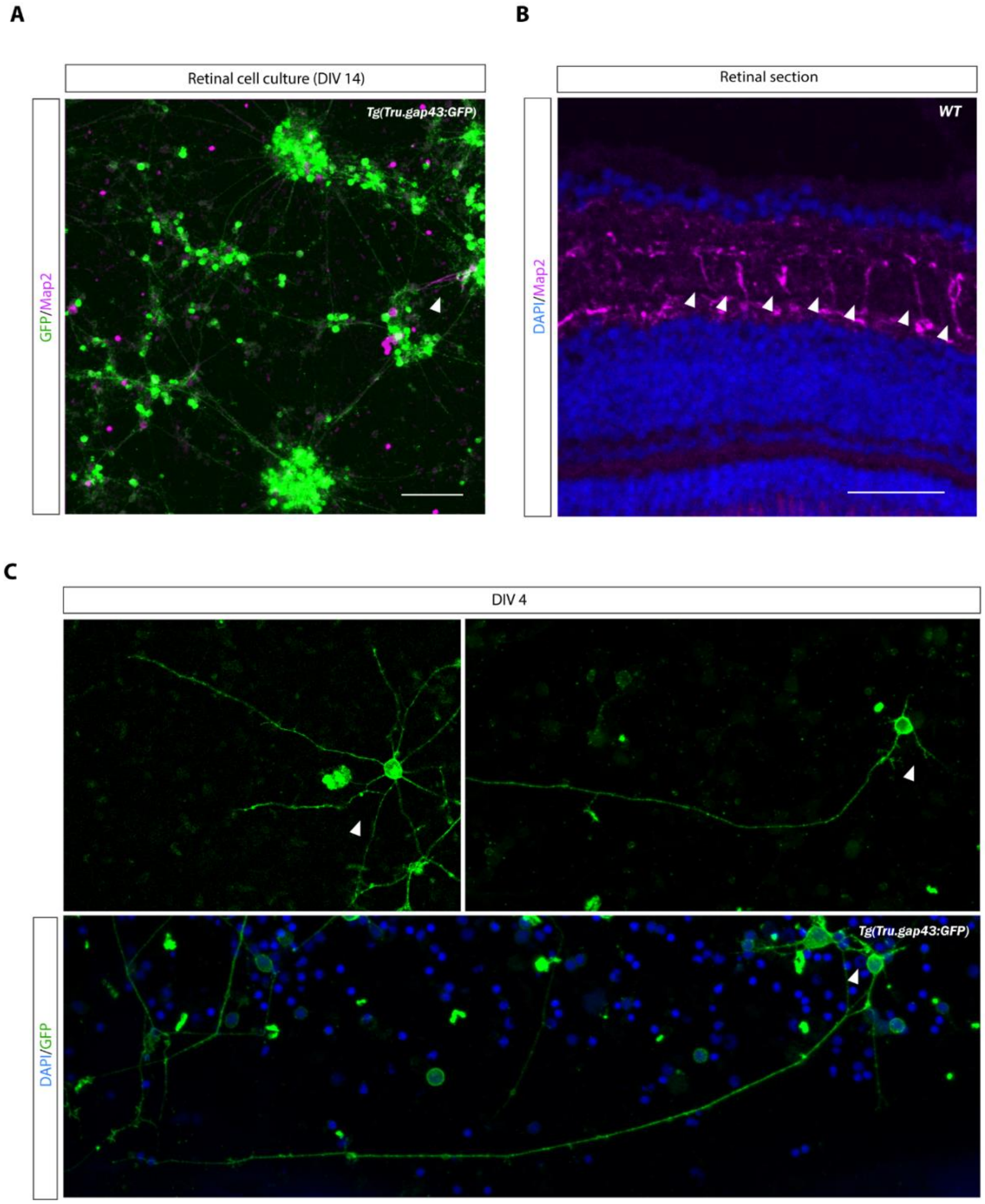

Supplementary Figure 5: Identification and characterization of dendrites in adult zebrafish retinal cell cultures and cryosections. (**A-B)** Dendrites cannot be reliably identified in cultured adult zebrafish retinal neurons using immunostainings for the dendritic marker Map2. Representative images of an adult *Tg(Tru.gap43:GFP)*^{mil1} (gap43) retinal cell culture at DIV 14 (A) and a naive adult wild type (WT) zebrafish retinal cryosection (B) stained for Map2 reveal that only a limited number of (RGC) dendrites in both the retinal cell culture and section are labeled (white arrowheads). This minimal Map2 expression is not representative for the total number of RGC dendrites present in the culture, nor in the inner retinal layers, and most likely, reflects differences in expression between individual cells. As such, we cannot rely on this marker to differentiate between dendrites and axons in our microfluidic setup. (**C)** Nevertheless, in mixed, sparsely labeled gap43/WT retinal cultures at DIV 4, individual RGCs can be observed with clear axon- and dendrite-like morphologies (white arrowheads). Scale bars: 100 µm (A), 50 µm (B,C). Div: *days in vitro*, MFD: microfluidic device, RGC: retinal ganglion cell.

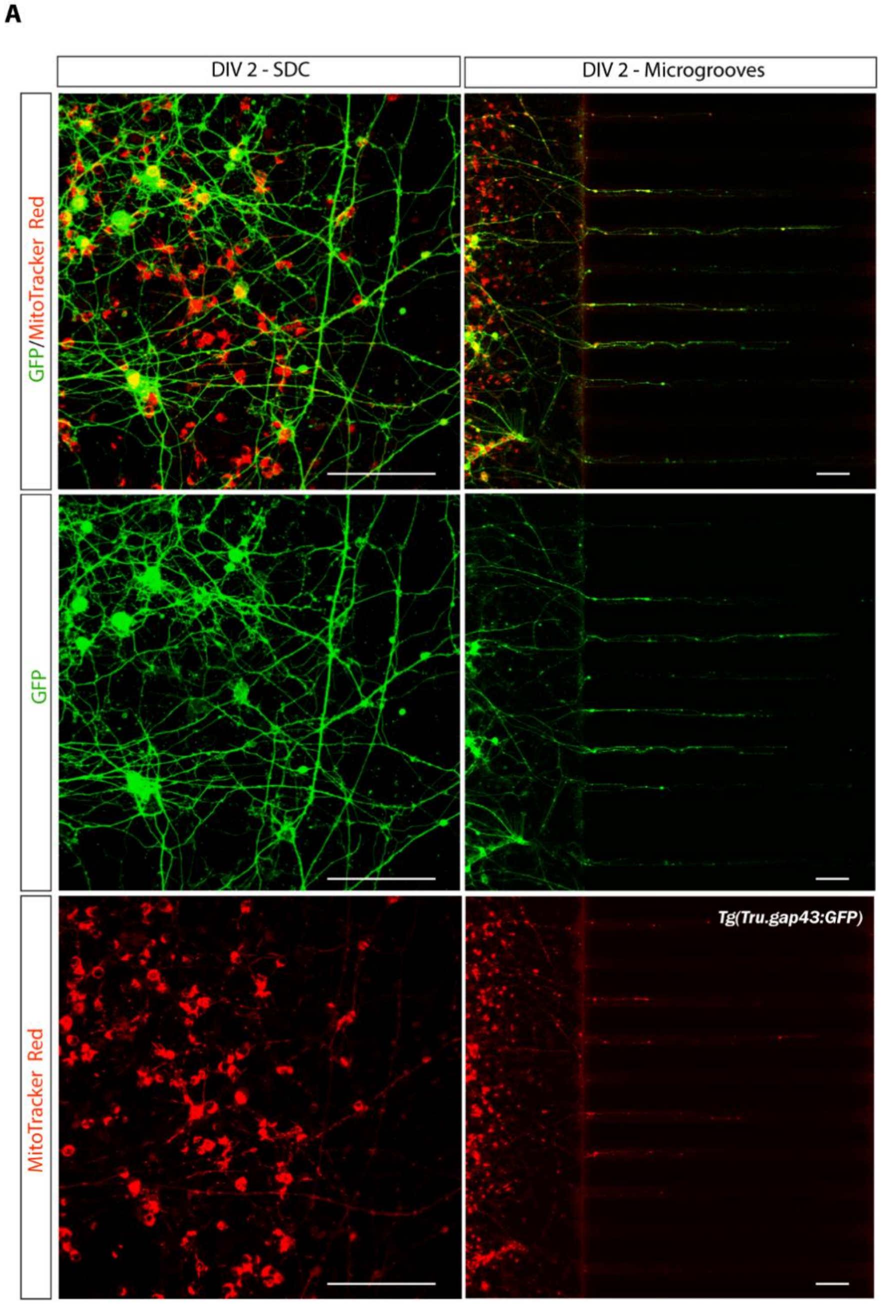
A
DIV 2 - SDC
DIV 2 - Microgrooves
GFP/MitoTracker Red
GFP
MitoTracker Red
Tg(Tru.gap43:GFP)

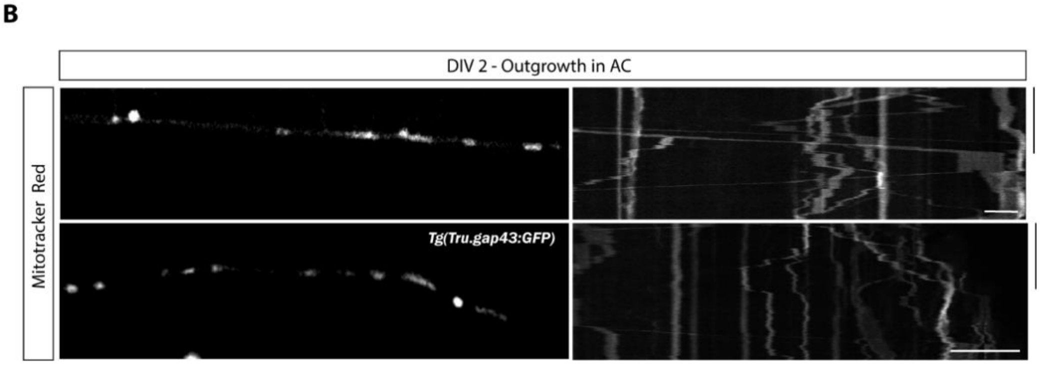
B
DIV 2 - Outgrowth in AC
Mitotracker Red
Tg(Tru.gap43:GFP)

Supplementary Figure 6: Visualization and characterization of mitochondrial motility in adult zebrafish retinal neurons using MitoTracker Red dye. **(A)** Mitochondria inside cultured adult zebrafish retinal neuron can be visualized by fluorescent labeling of mitochondria using MitoTracker Red dye. Live cell images taken at DIV 2 in *Tg(Tru.gap43:GFP)^mil1^* (gap43) retinal culture in an open compartment (SOC450) MFD indicate that the addition of MitoTracker Red enables visualization of mitochondria (bottom panels) inside RGC somata in the SDC, and inside axons growing into the microgrooves (middle panels). Of note, and in contrast to the data obtained using a mitochondrial-reporter line (see figure 9), all mitochondria of all retinal cells present in the culture are labeled. **(B)** Confocal still images of time-lapse live images taken during outgrowth (DIV 2), and resulting kymographs, demonstrate that mitochondrial mobility during adult zebrafish RGC outgrowth can be characterized using this mitochondrial labeling method. Kymographs are generated using ImageJ for 20 minutes with a 2-second interval. Scale bars: 50 µm (A), 10 µm (B). DIV: *days in vitro*, MFD: microfluidic device, RGC: retinal ganglion cell, SDC: somatodendritic compartment.

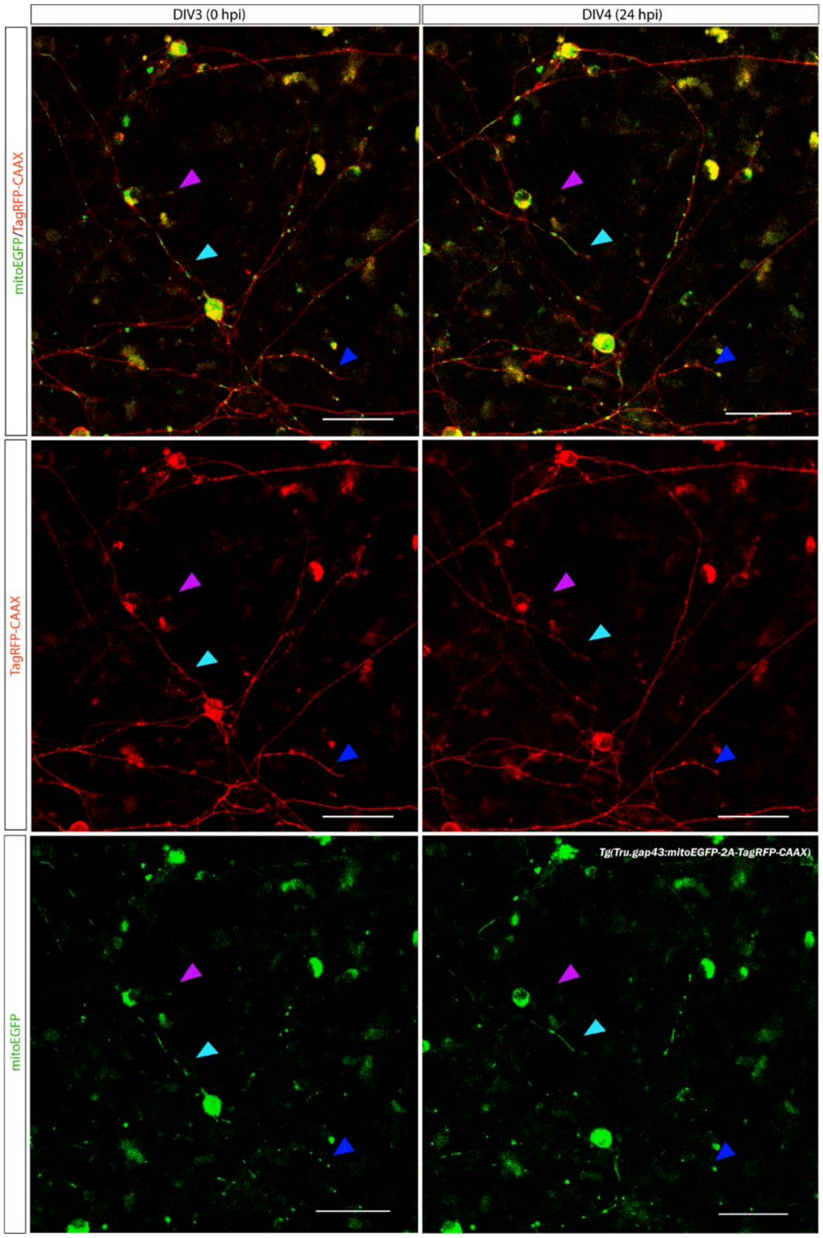

Supplementary Figure 7: Visualization of mitochondria in dendrite-like neurites of individual regenerating adult zebrafish RGCs. Pilot experiment demonstrating the future potential of a mixed *Tg(Tru.gap43:mitoEGFP-2A-tagRFP-CAAX)*^{ulb17}/wild type (gapmito/WT) culture to visualize and characterize dendrite-like neurite changes and mitochondrial dynamics concurrently during RGC axonal regeneration. Confocal live cell images taken immediately upon (DIV 3, 0 hpi), and 1 day after axotomy (DIV 4, 24 hpi) in a mixed gapmito/WT retinal culture in an open compartment (SOC450) MFD show changes in proximal dendrite-like neurites of isolated RGCs after injury (top panels). Colored arrowheads indicate the same neurites before and after regeneration and reveal changes in the localization of both neurites (middle panels) and mitochondria (bottom panels). Performing live-cell imaging in this model might enable to visualize these changes in real-time. Scale bars: 25 µm, DIV: days *in vitro*, RGC: retinal ganglion cell compartment. Scale bar: 50 µm. AC: axonal compartment, DIV: days *in vitro*; hpi: hours post injury, MFD: microfluidic device, RGC: retinal ganglion cell.

1.2 Supplementary movies

Supplementary movie 1: Axotomized axons are occasionally replaced by newly emerging neurites at DIV 3.
Confocal time-lapse live cell imaging of regenerating adult *Tg(Tru.gap43:GFP)*^{mil1} (gap43) zebrafish RGCs in a microfluidic setup taken immediately upon axotomy at DIV 3 (0-8 hpi), discloses that during axonal regrowth, newly outgrowing axons emerge in the microgrooves and AC. Of note, occasionally these newly outgrowing axons grow on top of regrowing or degenerating axons (yellow arrowhead), thereby complicating the identification of the latter on confocal still overview pictures. Time frame: 8 hours (30-minute interval, 3 fps).

Supplementary movie 2: Active movement of axonal growth cones during adult zebrafish RGC axonal outgrowth at DIV 2.
Confocal time-lapse live imaging of adult *Tg(Tru.gap43:GFP)*^{mil1} (gap43) zebrafish RGC axons, growing into the microgrooves of a microfluidic setup at DIV 2, illustrates how some axonal growth cones are very dynamic during outgrowth into the AC. As a result, registration of the complete diameter of these axons during subsequent (kymographic) analyses might be challenging. The same can be observed in time-lapse images of axonal regeneration at DIV 3. Time frame: 7 hours (30-minute interval, 3 fps).

Supplementary movie 3: Active migration of adult zebrafish retinal neurons at DIV 1.
Confocal time-lapse live imaging at DIV 1 in adult *Tg(Tru.gap43:GFP)*^{mil1} (gap43) retinal cultures demonstrates how outgrowing adult zebrafish RGCs are actively moving towards one another. As a result of this movement, clusters of cells will start to form during the following days in culture (figure 2). Time frame: 5 hours (10-minute interval, 5 fps).

Supplementary movie 4: Axonal outgrowth of adult zebrafish RGCs at DIV 2 in a microfluidic setup.
Confocal time-lapse live imaging of outgrowing adult *Tg(Tru.gap43:GFP)*^{mil1} (gap43) zebrafish RGCs in a MFD shows that numerous axons are entering and growing through the microgrooves at DIV 2, eventually reaching the AC. Time frame: 6 hours (30-minute interval, 2 fps).

Supplementary movie 5: Axonal regeneration of adult zebrafish RGCs at DIV 3 in a microfluidic setup.
Confocal time-lapse live imaging of adult *Tg(Tru.gap43:GFP)*^{mil1} (gap43) zebrafish RGCs at DIV 3 (0-8 hpi) discloses how RGC axons, axotomized at the border of the microgrooves and AC, spontaneously regrow into the AC. Some axotomized axons start to regenerate immediately after axotomy, while others only enter the AC after a few hours. In addition to regenerating (green arrowheads), static (red arrowheads) and degenerating (blue arrowheads) axons, also newly outgrowing axons (yellow arrowheads) can be observed in the AC. Time frame: 8 hours (30-minute interval, 4 fps).

Supplementary movie 6: Dendrite-like neurite remodeling during adult zebrafish RGC axonal regeneration **at DIV 3.**
Confocal time-lapse live imaging of regrowing adult zebrafish RGCs from mixed *Tg(Tru.gap43:GFP)*^{mil1} /wild type (gap43/WT) retinal cultures immediately after injury at DIV 3 (0-8

hpi) illustrates the potential of this sparsely labeled setup to visualize dendritic changes during axonal regeneration. Time frame: 2 hours (10-minute interval, 3 fps).

Supplementary movie 7: Mitochondrial mobility during axonal regeneration of adult zebrafish RGCs at DIV 3 in a microfluidic setup.
Confocal time-lapse live imaging of the mitochondrial mobility in regenerating adult zebrafish *Tg(Tru.gap43:mitoEGFP-2A-tagRFP-CAAX)*^{ulb17} (gapmito) RGCs at DIV 3 (5 hpi) reveals different actively regrowing axons with substantial mitochondrial mobility in both antero- and retrograde directions. Time frame: 25 minutes (5-second interval, 50fps).
